# The PTEN phosphatase functions cooperatively with the Fanconi anemia proteins in DNA crosslink repair

**DOI:** 10.1038/srep36439

**Published:** 2016-11-07

**Authors:** Elizabeth A. Vuono, Ananda Mukherjee, David A. Vierra, Morganne M. Adroved, Charlotte Hodson, Andrew J. Deans, Niall G. Howlett

**Affiliations:** 1 Department of Cell and Molecular Biology, University of Rhode Island, Kingston, Rhode Island, USA; 2 Department of Obstetrics, Gynecology, and Reproductive Biology, Michigan State University, Grand Rapids, Michigan, USA; 3 Genome Stability Unit, St. Vincent’s Institute, Fitzroy, VIC 3065, Australia; 4 Department of Medicine, The University of Melbourne, Parkville, VIC 3010, Australia

## Abstract

Fanconi anemia (FA) is a genetic disease characterized by bone marrow failure and increased cancer risk. The FA proteins function primarily in DNA interstrand crosslink (ICL) repair. Here, we have examined the role of the PTEN phosphatase in this process. We have established that PTEN-deficient cells, like FA cells, exhibit increased cytotoxicity, chromosome structural aberrations, and error-prone mutagenic DNA repair following exposure to ICL-inducing agents. The increased ICL sensitivity of PTEN-deficient cells is caused, in part, by elevated PLK1 kinase-mediated phosphorylation of FANCM, constitutive FANCM polyubiquitination and degradation, and the consequent inefficient assembly of the FA core complex, FANCD2, and FANCI into DNA repair foci. We also establish that PTEN function in ICL repair is dependent on its protein phosphatase activity and ability to be SUMOylated, yet is independent of its lipid phosphatase activity. Finally, *via* epistasis analysis, we demonstrate that PTEN and FANCD2 function cooperatively in ICL repair.

Fanconi anemia (FA) is a rare autosomal and X-linked disease characterized by congenital abnormalities, progressive pediatric bone marrow failure, and increased cancer risk in early adulthood^1^. FA is caused by mutation of any one of 21 genes (*FANCA, -B, -C, -D1/BRCA2, -D2, -E, -F, -G, -I, -J/BRIP1, -L, -M, -N/PALB2, -O/RAD51C, -P/SLX4, -Q/ERCC4, -R/RAD51, -S/BRCA1, -T/UBE2T,* -*U/XRCC2*, and *-V/REV7*). The FA proteins function in the FA-BRCA pathway to facilitate the removal of DNA interstrand crosslinks (ICLs) and to maintain genome stability^2^,^3^,^4^. ICLs are highly toxic lesions that covalently link DNA strands, imposing a direct physical block to DNA replication and RNA transcription. The FA-BRCA pathway resolves ICLs by first converting these lesions into DNA double-strand breaks (DSBs)^5^. The DSB is then repaired by homologous recombination (HR), a conservative DNA repair mechanism that uses the homologous DNA sequence on a sister chromatid or homologous chromosome as a template to copy and repair the damaged DNA. The RAD51 protein is the major cellular HR protein and catalyzes the critical strand invasion step of HR^6^,^7^.

Eight FA proteins, as well as several accessory proteins, form the FA core complex, a multisubunit E3 ubiquitin ligase that catalyzes the monoubiquitination of the FANCD2 and FANCI proteins^2^,^8^. FANCD2/I monoubiquitination is an essential step in the activation of the FA-BRCA pathway^8^,^9^. Upon DNA damage, monoubiquitinated FANCD2 co-localizes with several DNA repair proteins, including RAD51/FANCR and BRCA2/FANCD1, in discrete chromatin-associated nuclear foci^10^,^11^. FANCD2 and BRCA2/FANCD1 promote RAD51/FANCR nucleoprotein filament formation and DNA strand exchange during HR^10^,^12^,^13^.

In addition to being regulated by ubiquitination, the FA-BRCA pathway is also extensively regulated *via* phosphorylation. For example, FANCD2 and FANCI are phosphorylated by the two major DNA damage response kinases ATM (ataxia telangiectasia mutated) and ATR (ataxia telangiectasia and Rad3-related)^14^,^15^,^16^,^17^. FANCI phosphorylation on six clustered SQ/TQ motifs is required for its monoubiquitination and nuclear foci formation^16^. In addition, FANCM is hyperphosphorylated by PLK1 during mitosis, promoting its polyubiquitination and degradation by the proteasome^18^. Importantly, to date, no phosphatases have been directly linked to the FA-BRCA pathway.

*PTEN* encodes a dual specificity phosphatase capable of removing phosphates from both proteins and lipids^19^,^20^. The principal catalytic function of PTEN is to dephosphorylate the lipid second messenger phosphatidylinositol-3,4,5-triphosphate (PIP_3_), a potent activator of the AKT kinases^20^. Loss of PTEN catalytic function leads to de-repression of the phosphatidylinositol 3-kinase (PI3K)/AKT pathway and stimulation of cell growth and survival pathways^21^,^22^. While this plasma membrane-localized PTEN function is central to tumor suppression, recent studies have established that PTEN has PI3K/AKT-independent nuclear tumor suppressive functions^23^,^24^. Indeed, important roles for PTEN in the regulation of cell cycle progression and the maintenance of chromosome stability have recently been established^25^,^26^,^27^,^28^.

In this study, we have investigated the role of PTEN in ICL repair and in the regulation of the FA-BRCA pathway. We have established that PTEN plays an important role in ICL repair as PTEN-deficient cells, like FA patient cells, exhibit increased sensitivity to ICL-mediated cytotoxicity and display increased levels of chromosome structural aberrations following ICL exposure. The increased ICL sensitivity of PTEN-deficient cells is caused, in part, by elevated PLK1 kinase-mediated phosphorylation of FANCM, constitutive FANCM polyubiquitination and degradation, and the consequent inefficient assembly of the FA core complex, FANCD2, and FANCI into DNA repair foci. We also show that PTEN function in ICL repair is independent of its lipid phosphatase activity yet dependent on its protein phosphatase activity and its ability to be SUMOylated on K254. We also establish that PTEN deficiency leads to increased mutagenic ICL repair, exemplified by increased 53BP1 and DNA-PKcs-pS2056 nuclear foci formation, biomarkers of the error-prone nonhomologous DNA end joining (NHEJ) repair pathway. Finally, using an RNA interference approach in FA-D2 patient cells and PTEN-deficient tumor lines, we demonstrate that PTEN and FANCD2 function epistatically during ICL repair. Our results uncover important mechanistic insight into the role of nuclear PTEN in ICL repair and establish the convergence of two critical tumor suppressor pathways.

## Results

### PTEN is required for chromosome stability and cellular survival following mitomycin C treatment

**Figure 1:**
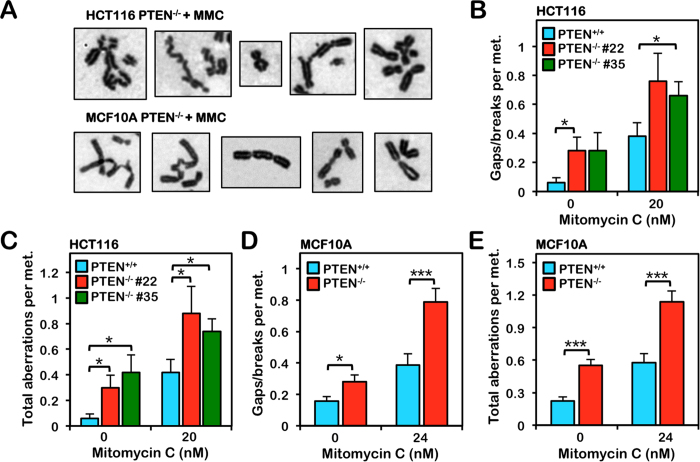
PTEN^−/−^ cells are hypersensitive to the clastogenic effects of mitomycin C. HCT116 and MCF10A PTEN^+/+^ and PTEN^−/−^ cells were incubated in the absence or presence of mitomycin C (MMC) for 24 h and metaphase spreads were analyzed for numerical and structural chromosome aberrations. (**A**) Representative images of the types of chromosome aberrations - including radial formations, telomere fusions, dicentrics, and complex aberrations - observed in PTEN^−/−^ cells following MMC treatment. (**B,C**) Quantification of chromosome gaps and breaks (**B**) and total chromosome aberrations (**C**) observed in HCT116 PTEN^+/+^ and two independent clones of PTEN^−/−^ cells incubated in the absence or presence of 20 nM MMC for 24 h. (**D,E**) Quantification of chromosome gaps and breaks (**D**) and total chromosome aberrations (**E**) observed in MCF10A PTEN^+/+^ and PTEN^−/−^ cells incubated in the absence or presence of 24 nM MMC for 24 h. *P < 0.05; **P < 0.01; ***P < 0.001.

To investigate the role of PTEN in ICL repair we treated isogenic HCT116 PTEN^+/+^ and PTEN^−/−^ cells with mitomycin C (MMC) and examined cellular cytotoxicity and metaphase chromosome aberrations. Similar to FA patient cells that are characteristically sensitive to ICL-inducing agents^29^,^30^ two independently derived PTEN^−/−^ lines exhibited increased sensitivity to MMC. The calculated LD_50_ values for PTEN^+/+^ cells were 2-fold greater than those for both PTEN^−/−^ lines (Figure S1A). PTEN^−/−^ cells also exhibited increased spontaneous and MMC-inducible chromosome gaps and breaks and complex aberrations, including radial formations (Fig. 1A–C). We next examined the role of PTEN in ICL repair in a non-transformed cell model using the isogenic mammary epithelial cells MCF10A PTEN^+/+^ and PTEN^−/−^. Again PTEN^−/−^ cells exhibited increased sensitivity to the cytotoxic effects of MMC (Figure S1B). We also observed an increased frequency of both spontaneous and MMC-inducible chromosome gaps and breaks and complex aberrations in the MCF10A PTEN^−/−^ cells compared to PTEN^+/+^ cells (Fig. 1A,D,E). MCF10A PTEN^−/−^ cells also exhibited a striking increase in both spontaneous and ICL-inducible centromere aberrations, exemplified by de-condensed centromeres, similar to that previously described^27^ (Figure S1C,D).

### PTEN is required for efficient MMC-inducible FANCD2 and FANCI nuclear foci formation

**Figure 2:**
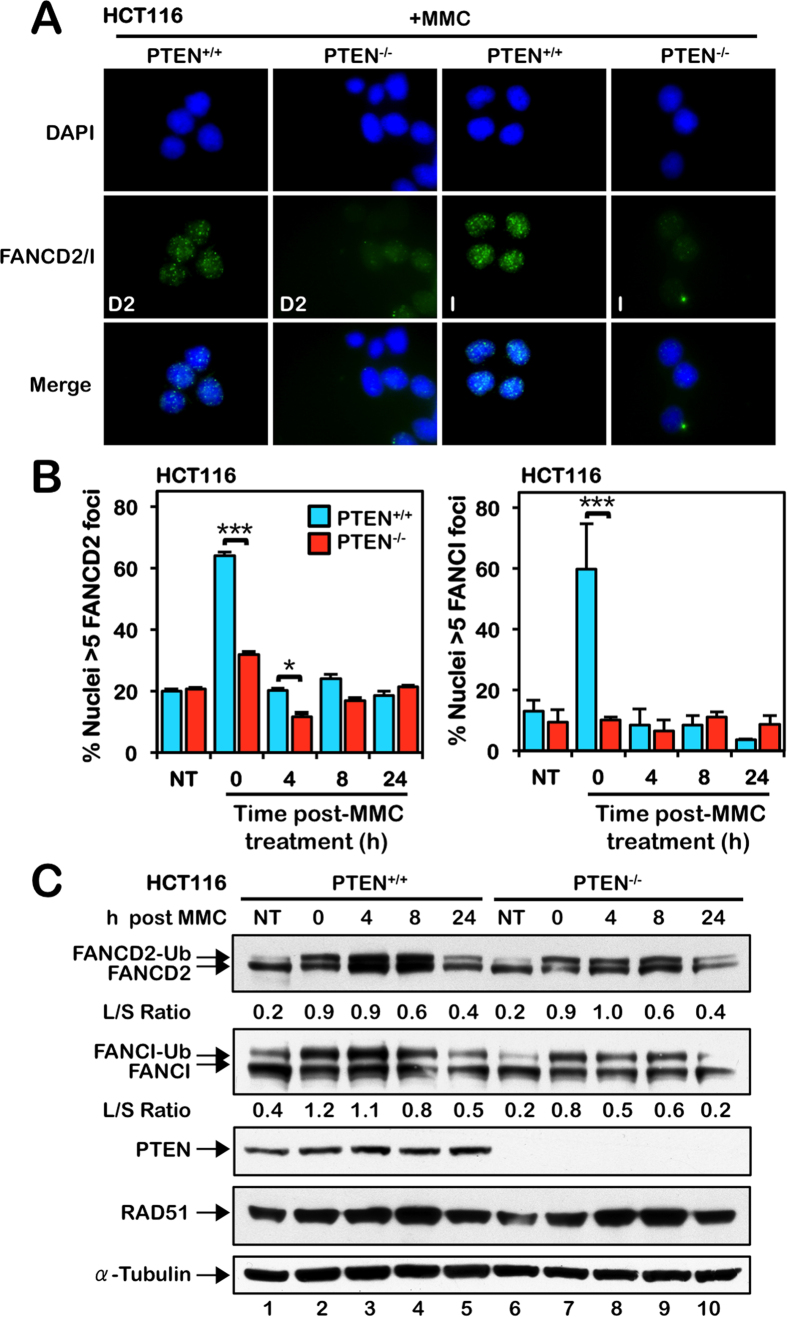
PTEN is required for efficient mitomycin C-inducible FANCD2 and FANCI nuclear foci formation. (**A**) HCT116 PTEN^+/+^ and PTEN^−/−^ cells were incubated in the absence or presence of 40 nM mitomycin C (MMC) for 18 h and FANCD2 and FANCI nuclear foci formation were analyzed by immunofluorescence microscopy. Representative images of FANCD2 and FANCI nuclear foci from cells exposed to MMC are shown. (**B**) Quantification of the percentage of PTEN^+/+^ and PTEN^−/−^ nuclei displaying greater than five discrete FANCD2 or FANCI nuclear foci. Cells were incubated in the absence (NT) or presence of 40 nM MMC for 18 h and allowed to recover for up to 24 h. *P < 0.05; **P < 0.01; ***P < 0.001. (**C**) Immunoblotting for FANCD2, FANCI, and RAD51 reveals no appreciable differences in levels of MMC-inducible FANCD2 and FANCI monoubiquitination or RAD51 between PTEN^+/+^ and PTEN^−/−^ cells. Cells were incubated in the absence or presence of 200 nM MMC for 24 h and allowed to recover for up to 24 h. To improve clarity and conciseness, the presented blots have been cropped. All gels were run under the same experimental conditions.

To gain insight into the molecular basis of the increased ICL sensitivity of PTEN-deficient cells, we examined the activation of the FA-BRCA pathway in these cells, a pathway known to play a critical role in the cellular ICL response^3^. Activation of this pathway occurs *via* the site-specific monoubiquitination of the FANCD2 and FANCI proteins and their assembly into discrete nuclear foci^9^,^31^,^32^. HCT116 PTEN^+/+^ and PTEN^−/−^ cells were treated with MMC for 16 h followed by a 24 h recovery period. Following MMC treatment, PTEN^+/+^ cells exhibited a strong increase in FANCD2 and FANCI nuclear foci formation with ~60% of cells displaying greater than 5 discrete nuclear foci (Fig. 2A,B). In contrast, no appreciable induction of FANCD2 or FANCI nuclear foci formation was observed for PTEN^−/−^ cells following MMC exposure (Fig. 2A,B). Re-expression of PTEN in PTEN^−/−^ cells restored efficient MMC-inducible FANCD2 and FANCI nuclear foci formation (see Fig. 6C,D). No overt differences in MMC-inducible FANCD2 or FANCI monoubiquitination were observed between PTEN^+/+^ and PTEN^−/−^ cells (Fig. 2C). Furthermore, using a cellular fractionation approach, we did not observe appreciable differences in the chromatin enrichment of FANCD2 or FANCI in the absence of PTEN (Figure S2A). Very similar results were obtained with MCF10A PTEN^−/−^ cells, which exhibited markedly reduced levels of MMC-inducible FANCD2 and FANCI nuclear foci, compared with PTEN^+/+^ cells (Figures S2B,C), but no discernible differences in MMC-inducible FANCD2 or FANCI monoubiquitination or chromatin enrichment (Figure S2D,E).

### Increased FANCM instability and defective chromatin recruitment of the FA core complex in PTEN-deficient cells

**Figure 3:**
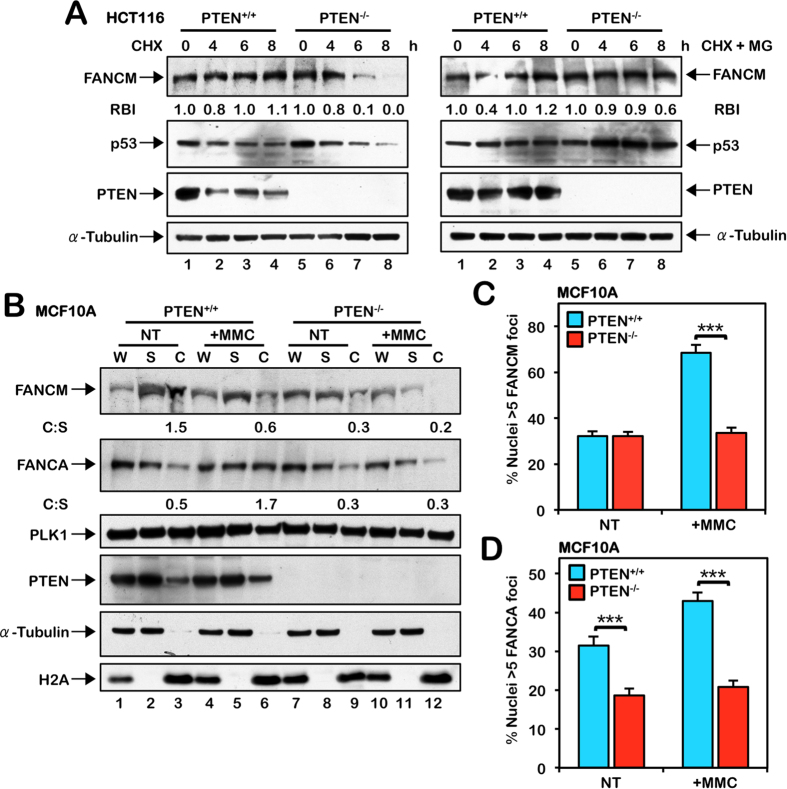
Increased FANCM instability and defective chromatin recruitment of the FA core complex in PTEN-deficient cells. (**A**) HCT116 PTEN^+/+^ and PTEN^−/−^ cells were incubated in the absence or presence of 40 μg/mL cycloheximide (CHX) alone or 40 μg/mL CHX and 4 μM MG132 (CHX + MG) for the indicated times. Whole-cell lysates were prepared and immunoblotted with anti-FANCM, anti-p53, anti-PTEN, and anti-α-tubulin antibodies. RBI, Relative protein band intensity with respect to the untreated sample lane. (**B**) Chromatin fractionation analysis of MCF10A PTEN^+/+^ and PTEN^−/−^ cells reveals a defect in the chromatin localization of FANCM and FANCA in the absence of PTEN. Cells were incubated in the absence (NT) or presence of 200 nM mitomycin C (MMC) for 24 h. W, unfractionated whole-cell lysate; S, soluble cytoplasmic and nuclear fraction; C, chromatin fraction. For (**A**,**B**), to improve clarity and conciseness, the presented blots have been cropped. All gels were run under the same experimental conditions. C:W, Ratio of protein in the chromatin fraction versus the whole-cell lysate. Immunoblotting experiments were performed multiple times with similar results. Protein band quantifications are from the immunoblots shown and are representative of results from several experiments. (**C**) Quantification of FANCM nuclear foci formation in MCF10A PTEN^+/+^ and PTEN^−/−^ cells reveals a defect in MMC-inducible FANCM nuclear foci formation in PTEN^−/−^ cells. Cells were incubated in the absence (NT) or presence of 200 nM MMC for 18 h. ***P < 0.001. (**D**) Quantification of FANCA nuclear foci formation in MCF10A PTEN^+/+^ and PTEN^−/−^ cells reveals reduced FANCA nuclear foci formation in PTEN^−/−^ cells both in the absence (NT) and presence of MMC. Cells were treated as described for (**C**). ***P < 0.001.

Previous studies have established that the FANCM protein is required for efficient recruitment of the FA core complex to chromatin and for the assembly of FANCD2 and FANCI nuclear foci^33^,^34^. Therefore we examined FANCM expression in PTEN^+/+^ and PTEN^−/−^ cells. In the absence of PTEN, we observed reduced levels of FANCM (Figure S3A,B). Modest reductions in total cellular levels of FANCA were also observed (Figure S3A,B). A cycloheximide pulse-chase experiment revealed that reduced FANCM levels were a consequence of increased FANCM turnover: increased FANCM instability could be rescued by inhibition of the proteasome with MG132 (Figs 3A and S3C). p53 was used a positive control for these experiments. Furthermore, we observed reduced chromatin-associated FANCM and ICL-inducible FANCM nuclear foci formation in the absence of PTEN (Figs 3B,C and S3D,G). Consistent with an important role for FANCM in promoting the chromatin localization of the FA core complex, we also observed defective ICL-inducible FANCA chromatin localization and nuclear foci formation in PTEN^−/−^ cells (Figs 3B,D and S3E,F,H).

### Inhibition of PLK1 rescues defective FANCD2 nuclear foci formation but is not sufficient to rescue the chromosome instability of PTEN^−/−^ cells

**Figure 4:**
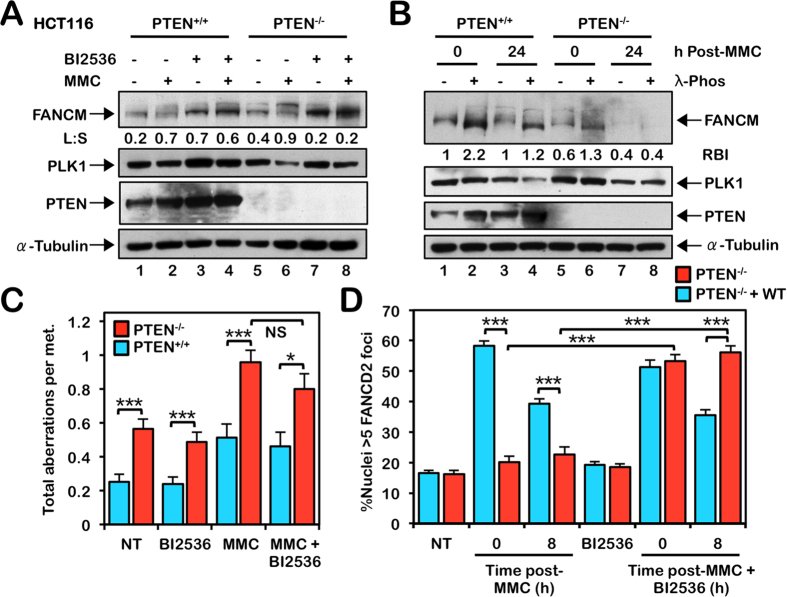
Inhibition of PLK1 rescues defective FANCD2 nuclear foci formation but is not sufficient to rescue the chromosome instability of PTEN^−/−^ cells. (**A**) HCT116 PTEN^+/+^ and PTEN^−/−^ cells were incubated in the absence (−) and presence (+) of 100 nM BI2536 and 500 nM mitomycin C (MMC) for 18 h, and whole-cell lysates were immunoblotted with the indicated antibodies. L:S, Ratio of phosphorylated to unphosphorylated FANCM. (B) PTEN^+/+^ and PTEN^−/−^ cells were incubated in the absence and presence of 200 nM MMC for 24 h. Whole-cell lysates were then incubated in the absence (−) or presence (+) of 10 U/μg λ-phosphatase for 4 h at 30 °C, followed by immunoblotting with the indicated antibodies. For (**A**,**B**), to improve clarity and conciseness, the presented blots have been cropped. All gels were run under the same experimental conditions. RBI, Relative unmodified FANCM protein band intensity. Immunoblotting experiments were performed multiple times with similar results. Protein band quantifications are from the immunoblots shown and are representative of results from several experiments. (**C**) PTEN^+/+^ and PTEN^−/−^ cells were incubated in the absence or presence of 2 nM BI2536, 20 nM MMC, or both BI2536 and MMC for 24 h and metaphase spreads were analyzed for the presence of numerical and structural chromosome aberrations. (**D**) PTEN^−/−^ cells stably expressing empty vector or wild-type PTEN were incubated in the absence or presence of 5 nM BI2536, 200 nM MMC, or both BI2536 and MMC for 24 h, and allowed to recover for 8 h, and FANCD2 nuclear foci formation were analyzed by immunofluorescence microscopy. *P < 0.05; ***P < 0.001.

Previous studies have established that nuclear PTEN stabilizes the APC/C (anaphase-promoting complex/cyclosome) E3 ubiquitin ligase complex and, in the absence of PTEN, there is an increase in the activity of APC/C substrates as they escape ubiquitin-mediated proteolysis^28^. One such APC/C target is the PLK1 mitotic kinase^28^. PLK1 phosphorylates FANCM and primes it for polyubiquitination and degradation during mitosis^18^. To determine if increased PLK1 activity contributes to the increased FANCM instability observed in PTEN^−/−^ cells, we incubated cells in the absence and presence of MMC and the small molecule PLK1 inhibitor BI2536^35^,^36^. We observed a marked stabilization of FANCM in both PTEN^+/+^ and PTEN^−/−^ cells following BI2536 treatment, stabilization being more pronounced in PTEN^−/−^ cells (Fig. 4A). Furthermore, higher molecular weight FANCM isoforms, which are consistently more evident in PTEN^−/−^ cells (Fig. 4A,B, compare lanes 1 and 5), become reduced upon BI2536 treatment (Fig. 4A). A λ-phosphatase assay indicates that these higher molecular weight FANCM isoforms most likely represent both phosphorylated and polyubiquitinated FANCM, consistent with previous findings^18^, and that FANCM is particularly unstable in PTEN^−/−^ cells following MMC exposure (Fig. 4B). To determine if PLK1 inhibition could rescue the ICL sensitivity of PTEN^−/−^ cells, we co-treated cells with MMC and BI2536 and examined metaphase spreads for chromosome aberrations. While BI2536 treatment resulted in a slight reduction in levels of ICL-inducible chromosome aberrations in PTEN^−/−^ cells, this decrease was not statistically significant at the concentrations tested (Fig. 4C). Higher concentrations of BI2536 lead to M-phase arrest precluding metaphase chromosome analyses. BI2536-mediated PLK1 inhibition did, however, rescue the defective MMC-inducible FANCD2 nuclear foci formation in PTEN^−/−^ cells expressing empty vector (Fig. 4D). Importantly, 8 h following exposure to MMC and BI2536, while the percentage of PTEN^+/+^ nuclei exhibiting FANCD2 nuclear foci had returned to pre-exposure levels, FANCD2 nuclear foci persisted in PTEN^−/−^ cells (Fig. 4D). As increased FANCM phosphorylation in PTEN^−/−^ cells could be a consequence of increased PLK1 kinase activity or decreased PTEN phosphatase activity, we performed an *in vitro* kinase and phosphatase assay with purified FANCM, PLK1, and PTEN. While we observed robust PLK1-mediated FANCM phosphorylation, under the conditions tested, we did not observe any appreciable decrease in levels of phosphorylated FANCM upon incubation with PTEN (Figure S4). Taken together, these results indicate that, in the absence of PTEN, elevated PLK1 activity contributes to increased FANCM proteolysis leading to inefficient chromatin recruitment of the FA core complex and attenuated FANCD2 nuclear foci formation.

### Increased γH2AX, 53BP1 and DNA-PKcs pS2056 nuclear foci formation in PTEN-deficient cells

**Figure 5:**
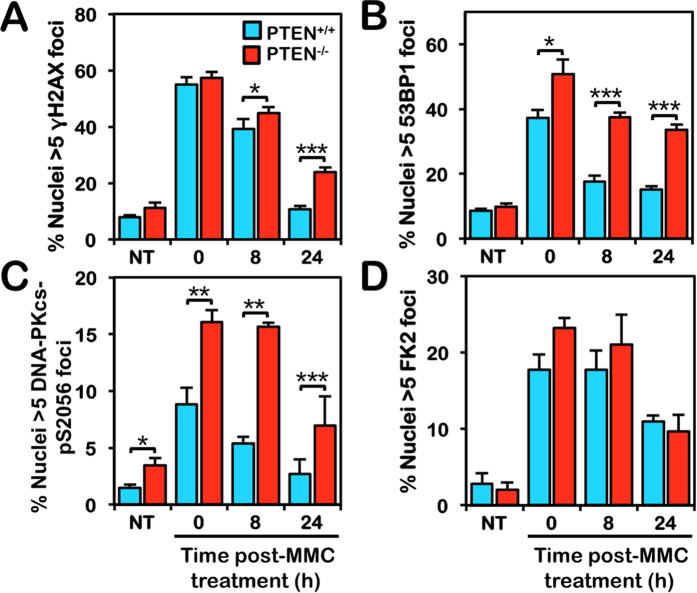
Increased γH2AX, 53BP1, and DNA-PKcs pS2056 nuclear foci formation in PTEN-deficient cells. (**A**) Quantification of γH2AX nuclear foci formation in HCT116 PTEN^+/+^ and PTEN^−/−^ cells reveals persistent elevated levels of γH2AX nuclear foci in PTEN^−/−^ cells following mitomycin C (MMC) treatment. Cells were incubated in the absence (NT) or presence of 40 nM MMC for 18 h and allowed to recover for up to 24 h. ***P < 0.001. (**B**) Quantification of 53BP1 nuclear foci formation in PTEN^+/+^ and PTEN^−/−^ cells reveals persistently elevated levels of 53BP1 nuclear foci in PTEN^−/−^ cells following MMC treatment. Cells were treated as described for (**A**). (**C**) Quantification of DNA-PKcs pS2056 nuclear foci formation in PTEN^+/+^ and PTEN^−/−^ cells reveals persistently elevated levels of DNA-PKcs pS2056 nuclear foci in PTEN^−/−^ cells. Cells were treated as described for (**A** and **B**). (**D**) Quantification of FK2 nuclear foci formation reveals no overt differences in levels of mono-, multi-, or poly-ubiquitin conjugates between PTEN^+/+^ and PTEN^−/−^ cells. Cells were treated as described for (**A–C**). *P < 0.05; **P < 0.01; ***P < 0.001.

To gain further insight into the underlying mechanisms of chromosome instability in PTEN-deficient cells, we next examined the levels of several DNA damage response biomarkers in PTEN^+/+^ and PTEN^−/−^ cells using immunofluorescence microscopy. Cells were treated with MMC for one cell cycle and allowed to recover for up to 24 h following exposure. We observed a large increase in γH2AX nuclear foci formation in both PTEN^+/+^ and PTEN^−/−^ cells following MMC exposure (Fig. 5A). Following 24 h recovery, the percentage of PTEN^+/+^ nuclei exhibiting γH2AX nuclear foci had returned to pre-exposure levels. However, in contrast, γH2AX nuclear foci persisted in PTEN^−/−^ nuclei at this time point (Fig. 5A). We also observed a large increase in 53BP1 nuclear foci formation in both PTEN^+/+^ and PTEN^−/−^ cells following MMC exposure (Fig. 5B). However, persistently elevated levels of PTEN^−/−^ nuclei staining positive for 53BP1 nuclear foci formation were observed for up to 24 h following MMC treatment (Fig. 5B). Similar findings of increased and/or persistent γH2AX and 53BP1 nuclear foci were observed for MCF10A PTEN^−/−^ cells (Figure S5A,B). We also observed increased levels of chromatin-associated 53BP1 in PTEN^−/−^ cells in the absence and presence of MMC (Figure S5C). Next we examined DNA-PKcs pS2056 nuclear foci formation, a biomarker of NHEJ^37^,^38^, in PTEN^+/+^ and PTEN^−/−^ cells. Similar to γH2AX and 53BP1, persistently elevated levels of DNA-PKcs pS2056 nuclear foci formation were observed in PTEN^−/−^ nuclei at all time points following MMC treatment (Fig. 5C). In contrast, no differences in overall levels of mono-, multi-, or poly-ubiquitin conjugates between PTEN^+/+^ and PTEN^−/−^ cells were observed (Fig. 5D). Collectively, these results strongly suggest that PTEN-deficient cells have a defect in efficient DSB repair and preferentially use mutagenic DSB repair pathways such as NHEJ. Notably, we did not observe any significant differences in levels of RAD51 protein expression (Figs 2C and S2D), chromatin enrichment (Figure S2A,E), or nuclear foci formation (Figure S5D,E), between PTEN^+/+^ and PTEN^−/−^ cells for both HCT116 and MCF10A cell models.

### PTEN function in ICL repair is protein phosphatase and SUMOylation-dependent

**Figure 6:**
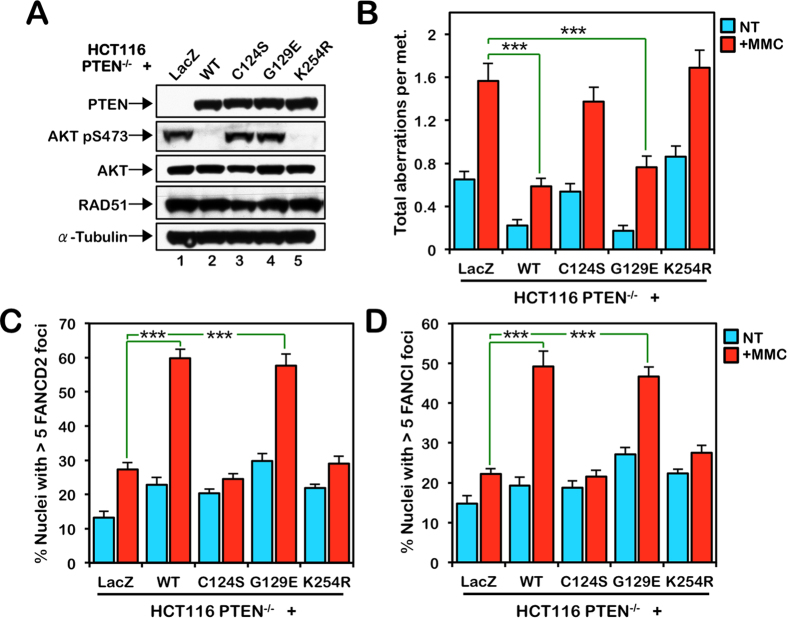
PTEN function in ICL repair is protein phosphatase and SUMOylation-dependent. (**A**) HCT116 PTEN^−/−^ cells were stably transduced with pLenti6.2-LacZ, -PTEN-WT, -PTEN-C124S, -PTEN-G129E, and -PTEN-K254R. Whole-cell lysates were prepared and immunoblotted for PTEN, AKT, AKT pS473, RAD51, and α-tubulin. To improve clarity and conciseness, the presented blots have been cropped. All gels were run under the same experimental conditions. (**B**) PTEN-C124S and PTEN-K254R fail to rescue the sensitivity of PTEN^−/−^ cells to the clastogenic effects of mitomycin C (MMC), in contrast to PTEN-WT or PTEN-G129E. Cells were incubated in the absence (NT) or presence of 20 nM MMC for 24 h and metaphase spreads were analyzed for structural chromosome aberrations. ***P < 0.001. (**C,D**) Defective FANCD2 (**C**) and FANCI (**D**) nuclear foci formation in PTEN^−/−^ cells expressing LacZ, PTEN-C124S, and PTEN-K254R. Cells were incubated in the absence (NT) or presence of 200 nM MMC for 18 h. ***P < 0.001.

To gain further mechanistic insight into the role of PTEN in ICL repair, we complemented HCT116 PTEN^−/−^ cells with wild type PTEN, lipid phosphatase-defective PTEN-G129E, lipid and protein phosphatase-defective PTEN-C124S, and SUMOylation-incompetent PTEN-K254R^19^,^25^,^39^. The PTEN variants behaved as predicted with only wild type and PTEN-K254R able to down-regulate the PI3K/AKT pathway, as demonstrated by loss of AKT-pS473 signal (Fig. 6A). We exposed these cells to MMC and analyzed metaphase chromosomes for the presence of gaps, breaks, and radial formations. Both wild type PTEN and the PTEN-G129E mutant rescued the sensitivity of PTEN^−/−^ cells to MMC-induced chromosome damage. However, PTEN-C124S and PTEN-K254R failed to rescue the ICL sensitivity of PTEN^−/−^ cells (Fig. 6B). Cells stably expressing PTEN-C124S and PTEN-K254R exhibited increased chromosome gaps and breaks and radial formations compared with cells expressing wild-type PTEN, following exposure to MMC (Fig. 6B). Furthermore, analysis of FANCD2 and FANCI nuclear foci formation in these cells revealed inefficient MMC-inducible FANCD2 and FANCI nuclear foci formation in PTEN^−/−^ cells stably expressing PTEN-C124S and PTEN-K254R, similar to cells expressing LacZ (Fig. 6C,D). To further confirm the requirement for PTEN protein (but not lipid) phosphatase activity, we also show that PTEN^−/−^ cells stably expressing PTEN-Y138L are ICL sensitive and fail to properly form FANCD2 and FANCI nuclear foci formation (Figure S6A–D). This missense mutation was previously shown to abrogate PTEN protein phosphatase activity but not lipid phosphatase activity^40^. Moreover, while wild type PTEN and the PTEN-G129E mutant rescued defective ICL-inducible FANCA and FANCM nuclear foci formation, the PTEN-C124S, -K254R, and Y138L mutants did not (Figure S6E,F). Recent studies have suggested that PTEN SUMOylation promotes its nuclear export^25^. Consistent with these findings, we observed increased PTEN-K254R stability and less efficient removal of PTEN-K254R from chromatin following ICL exposure, compared with wild type PTEN (Figure S7). Taken together, these results establish that PTEN function in the activation of the FA pathway and ICL repair is lipid phosphatase-independent, yet dependent on its protein phosphatase activity and ability to undergo SUMOylation on K254.

### PTEN and FANCD2 epistasis analysis

**Figure 7:**
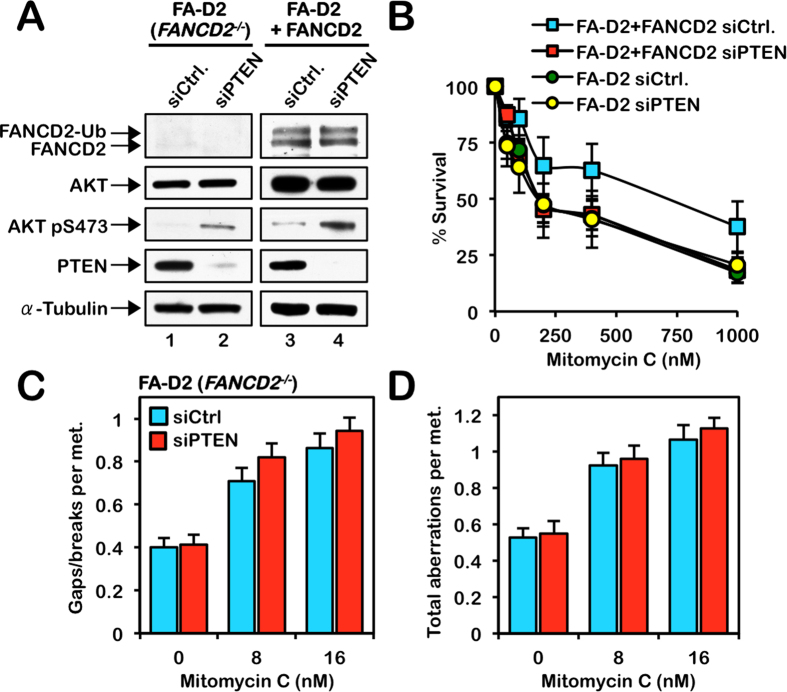
PTEN functions epistatically with FANCD2 in ICL repair. (**A**) siRNA-mediated knockdown of PTEN in FA-D2 (*FANCD2*^−/−^) and FA-D2 + FANCD2 cells. Increased levels of AKT S473 phosphorylation confirmed the functional depletion of PTEN. To improve clarity and conciseness, the presented blots have been cropped. All gels were run under the same experimental conditions. (**B**) Knockdown of PTEN (siPTEN) in FA-D2 + FANCD2 cells leads to increased sensitivity to mitomycin C (MMC) cytotoxicity. In contrast, knockdown of PTEN in FA-D2 (*FANCD2*^−/−^) cells does not lead to a further increase in sensitivity to MMC cytotoxicity. siCtrl, non-targeting siRNA. (**C** and **D**) Metaphase chromosome analysis of FA-D2 (*FANCD2*^−/−^) cells reveals that knockdown of PTEN does not lead to a further increase in the levels of chromosome gaps and breaks (**C**) or total chromosome aberrations (**D**), including radial formations, dicentrics, and complex aberrations.

To determine if PTEN functions epistatically with the FA proteins in ICL repair, we used an RNA interference approach to deplete PTEN in FA-D2 (*FANCD2*^−/−^) patient-derived cells stably expressing empty vector or pLenti6.2-FANCD2 (FA-D2 + FANCD2), and examined their response to MMC. Increased levels of AKT S473 phosphorylation confirmed the functional depletion of PTEN in FA-D2 and FA-D2 + FANCD2 cells (Fig. 7A). Consistent with our earlier findings demonstrating an important role for PTEN in ICL repair, depletion of PTEN in FA-D2 + FANCD2 cells resulted in increased sensitivity to the cytotoxic effects of MMC (Fig. 7B). However, no differences in sensitivity to MMC cytotoxicity were observed between FA-D2 cells transfected with control non-targeting siRNA or PTEN siRNA, or FA-D2 + FANCD2 cells transfected with PTEN siRNA (Fig. 7B). Similarly, depletion of PTEN in FA-D2 cells did not lead to a further increase in the numbers of chromosome gaps, breaks or complex aberrations (Figs 7C,D and S8A). We also performed the reciprocal analyses whereby we knocked down FANCD2 in the PTEN-deficient prostate carcinoma cell line PC-3 again using siRNA (Figure S8B). No increase in MMC-sensitivity was observed upon knockdown of FANCD2 in PC-3 cells (Figure S8C). Similarly, depletion of FANCD2 in PC-3 cells did not lead to an increase in chromosome gaps, breaks or complex aberrations (Figure S8D–F). Collectively, these results strongly suggest that FANCD2 and PTEN function epistatically in the cellular ICL response.

## Discussion

*PTEN* is one of the most widely mutated genes in cancer^41^,^42^,^43^. While a major tumor suppressive function of PTEN is the regulation of PI3K/AKT signaling, several recent studies have indicated an important PI3K/AKT-independent function for PTEN in the maintenance of chromosome stability^25^,^27^,^44^. However, this function remains poorly defined. Here, we have expanded our understanding of the function and regulation of PTEN in the maintenance of chromosome stability by specifically analyzing the role of PTEN in ICL repair. We demonstrate that cells lacking PTEN are phenotypically similar to cells from FA patients and exhibit increased ICL-sensitivity. We also establish a novel requirement for PTEN in the activation of the FA-BRCA pathway: PTEN is necessary for efficient ICL-inducible FANCD2 and FANCI nuclear foci formation. In contrast, ICL-inducible FANCD2 and FANCI monoubiquitination and chromatin enrichment are intact in the absence of PTEN. The uncoupling of monoubiquitination and chromatin enrichment from nuclear foci formation and effective ICL repair has previously been observed: *Usp1*^−/−^ murine embryonic fibroblasts display elevated levels of monoubiquitinated Fancd2 and Fancd2 chromatin localization, but fail to support Fancd2 nuclear foci formation and exhibit ICL-hypersensitivity^45^. Similarly, Fancm and Fancs/Brca1 knockout mouse cells show Fanci and Fancd2 monoubiquitination after high doses of DNA damage, but do not properly localize these proteins into DNA damage foci^46^,^47^. These findings emphasize that the assembly of FANCD2 and FANCI into nuclear foci is a critical determinant of efficient ICL repair. It is highly likely that the targeting of FANCD2 and FANCI to specific genomic loci, for example actively transcribed regions where co-transcriptional RNA-DNA hybrids (R-loops) arise, is essential for the promotion of efficient ICL repair^48^,^49^. Indeed, ongoing studies in our laboratory indicate that targeting to specific histone methyl marks is key for the assembly of FANCD2 and FANCI nuclear foci and efficient ICL repair (Paquin, K.L. and Howlett, N.G., unpublished findings).

Here, we also provide novel mechanistic insight into the role of PTEN in the promotion of efficient ICL repair (Figure S9). We establish a requirement for PTEN protein phosphatase activity, but not its lipid phosphatase activity, in ICL repair. PTEN has recently been shown to dephosphorylate MCM2 S41 and PLK1 T210^50^,^51^, and both of these mechanisms are likely to contribute to the observed phenotypes of PTEN^−/−^ cells. Dephosphorylation of MCM2 S41 prevents replication fork progression under conditions of replication stress^50^. ICLs are well known to pose a potent block to replication fork progression^52^. PLK1 T210 dephosphorylation leads to its deactivation and destabilization^51^. As PLK1 phosphorylates FANCM during mitosis and promotes its polyubiquitination and degradation by the proteasome^18^, a failure to deactivate PLK1 would be expected to lead to constitutive FANCM polyubiquitination and degradation. Nuclear PTEN has also been shown to promote the assembly of the APC/C-CDH1 ubiquitin ligase complex^28^. In the absence of PTEN, APC/C-CDH1 substrates, such as PLK1 and Aurora A kinase escape ubiquitin-mediated proteolysis and remain active^28^. In our study, we demonstrate that FANCM protein instability and FANCD2 nuclear foci formation can be rescued by inhibition of the proteasome with MG132 and by inhibition of PLK1 with BI2536, respectively. Collectively, our results support a model whereby, in the absence of PTEN, constitutive PLK1 activity leads to constitutive FANCM polyubiquitination and degradation, precluding efficient activation of the FA pathway (Figure S9). It is also important to note that the relationship between PTEN and PLK1 is complex; in addition to PTEN dephosphorylating PLK1^51^ and promoting the assembly of the APC/C-CDH1 complex^28^, PLK1 directly phosphorylates PTEN on S380 promoting its accumulation in chromatin^53^.

In this study, we have also established a requirement for PTEN SUMOylation on K254 in ICL repair. PTEN-K254R fails to restore efficient FANCD2 and FANCI nuclear foci formation and ICL repair in PTEN^−/−^ cells. These findings are consistent with a recent study describing a role for PTEN SUMOylation in the cellular response to ionizing radiation^25^. PTEN SUMOylation is thought to promote its nuclear export, as the inhibition of nuclear export with leptomycin B was shown to result in an accumulation of PTEN-K254R in the nucleus^25^, which is also consistent with our findings. Nevertheless, the role of PTEN SUMOylation in the DNA damage response remains to be clearly determined. Several key DNA damage response proteins, including MDC1 and RPA1, are targeted for proteasome-mediated degradation *via* SUMOylation and subsequent polyubiquitination by the RNF4 SUMO-targeted E3 ubiquitin ligase (STUbL)^54^,^55^. In the case of RPA1, its targeted removal from resected single-stranded DNA facilitates RAD51 nucleoprotein filament formation prior to the assembly of the synaptonemal complex during HR repair^56^. Upon dephosphorylating one or more key substrates, PTEN SUMOylation and its targeted degradation may be necessary to facilitate subsequent DNA repair steps or enable replication fork resumption following removal of the ICL block.

Finally, using FA-D2 patient cells, PTEN-deficient tumor lines, and siRNA targeting FANCD2 and PTEN, we have also established that PTEN and FANCD2 function epistatically during the process of ICL repair, as the combined loss of both proteins conferred no greater sensitivity to the cytotoxic or clastogenic effects of MMC. These findings are consistent with PTEN functioning upstream of FANCD2 and FANCI activation. Similar to FA patient cells, we have also established that error-prone DNA repair pathways are preferentially activated in the absence of PTEN^37^,^57^. PTEN-deficient cells display elevated and persistent levels of 53BP1 and DNA-PKcs pS2056 nuclear foci following ICL exposure, indicative of increased usage of the typically mutagenic NHEJ DNA repair pathway. Persistent 53BP1 nuclear foci have also been observed in PTEN^−/−^ cells exposed to ionizing radiation^25^. The defect in the timely dissolution of γH2AX nuclear foci formation and the increased incidence of chromosome structural aberrations in PTEN^−/−^ cells observed in our study are also indicative of an increased reliance on NHEJ. The reasons for NHEJ bias in FA and PTEN-deficient cells remain to be determined. A previous study had established that PTEN and the E2F-1 transcription factor cooperate to regulate the transcription of RAD51^27^. As DSBs can be repaired *via* HR or NHEJ, the increased NHEJ bias in PTEN-deficient cells may be a direct consequence of compromised HR due to reduced RAD51 protein levels^27^. However, consistent with the findings of others^58^, we did not observe appreciable differences in RAD51 expression, chromatin enrichment, or nuclear foci formation between PTEN^+/+^ and PTEN^−/−^ cells. Elegant studies on ICL repair using *Xenopus* egg extracts have recently established that RAD51 ICL binding occurs upstream of DSB formation and is not affected by the absence of FANCD2 or FANCI, indicating that they are most likely not required for RAD51 chromatin loading^59^. FANCD2 is, however, necessary for the dual incisions flanking the ICL that occur prior to DSB formation^60^. In the absence of PTEN, inefficient FANCD2 and FANCI nuclear foci formation most likely leads to a failure to generate these incisions resulting in the subversion of repair from error-free HR to error-prone NHEJ. Accordingly, several studies have established an important role for PTEN in HR^25^,^61^. As HR-deficient tumors are hypersensitive to inhibitors of poly (ADP-ribose)-polymerase-1 (PARP)^62^,^63^, our studies, and those of others, continue to raise the prospect of tailored combination chemo- and radiation therapeutic approaches for *PTEN-*deficient tumors.

## Materials and Methods

### Cell culture

HCT116 PTEN^+/+^, PTEN^−/−^ #22, and PTEN^−/−^ #35^64^ (a kind gift from Todd Waldman, Lombardi Cancer Center, Georgetown University) were grown in McCoy’s medium supplemented with 12% v/v FBS, 1% v/v L-glutamine, and 1% v/v penicillin/streptomycin. Stably complemented HCT116 lines were generated by transduction with pLenti6.2/V5-DEST (Invitrogen) lentivirus harboring wild type or mutant *PTEN* cDNAs. Stable cell lines were grown in McCoy’s medium supplemented with 5 μg/ml blasticidin. FA-D2 (*FANCD2*^*hy/−*^) cells were purchased from Coriell Cell Repositories (Catalog ID GM16633)^65^. Corrected FA-D2 cells were generated by transduction with pLenti6.2-FANCD2 lentivirus, followed by selection in DMEM supplemented with 15% v/v FBS, 1% v/v L-glutamine, 1% v/v penicillin/streptomycin and 2 μg/ml blasticidin. The PC-3 prostate adenocarcinoma line (a kind gift from David Mills, Department of Medicine, Brown University) was grown in DMEM supplemented with 12% v/v FBS, 1% v/v L-glutamine, and 1% v/v penicillin/streptomycin. MCF10A PTEN^+/+^ and PTEN^−/−^ cells (Horizon Discovery, U.K.) were grown in DMEM F12 supplemented with 5% v/v horse serum, 20 ng/ml epidermal growth factor, 0.5 mg/ml hydrocortisone, 100 ng/ml cholera toxin, 10 μg/ml insulin, 1% v/v L-glutamine, and 1% v/v penicillin/streptomycin. Detailed descriptions of plasmids, site-directed mutagenesis, siRNA transfections, and chromosome breakage, cytotoxicity, and cell proliferation assays are included in the Supplemental Experimental Procedures.

### Immunoblotting and antibodies

For immunoblotting analysis, cell suspensions were washed in ice-cold PBS and lysed in 50 mM Tris.Cl pH 7.4, 1.0% v/v NP-40, 0.25% w/v Deoxycholic acid, 150 mM NaCl, 1 mM EGTA, 1 mM PMSF, 1 mM Na_3_O_4_V, 1 mM NaF, plus protease inhibitors cocktail (Roche). The cellular fractionation method is described in the Supplemental Experimental Procedures. Proteins were resolved on NuPAGE 3–8% w/v Tris-Acetate or 4–12% w/v Bis-Tris gels (Invitrogen) and transferred to polyvinylidene difluoride (PVDF) membranes. The following antibodies were used: rabbit polyclonal anti-53BP1 (sc-22760; Santa Cruz Biotechnology), anti-FANCA (ABP6201; Cascade), anti-FANCD2 (NB100–182; Novus Biologicals), anti-FANCI (Dr. Patrick Sung, Yale University and A301-254A; Bethyl Laboratories), anti-FANCM (a kind gift from Dr. Ruhikanta Meetei, Cincinnati Children’s Hospital), anti-FANCM (3821; Fanconi Anemia Research Fund), anti-H2A (07–146; Millipore), and anti-PTEN (9559;Cell Signaling), and mouse monoclonal anti-FANCM (CP3.2, CV11.1, and CV5.1), anti-γH2AX (05–636;Millipore), anti-PTEN (6H2.1;Cascade), anti-RAD51 (sc-8349; Santa Cruz), and anti-α-tubulin (MS-581-PO; Lab Vision).

### Immunofluorescence microscopy

For immunofluorescence (IF) analyses, cells were seeded in four-well tissue culture slides (BD Biosciences) or on cover slips (Corning) and treated with MMC for 18 h. Cells were fixed in 4% w/v paraformaldehyde in PBS for 15 min on ice, followed by permeabilization for 5 min in 0.3% v/v Triton X-100 in PBS. Fixed cells were incubated with primary antibodies in 5% v/v goat serum, 0.1% v/v NP40, in PBS for 1 h, washed three times with PBS and then incubated with Alexafluor 488-conjugated anti-mouse or anti-rabbit secondary antibodies (Invitrogen) for 45 min. Cells were then counterstained and mounted in vectashield plus 406-diamidine-2-phenylindole dihydrochloride (DAPI) (Vector Laboratories) and visualized using a Zeiss AxioImager.A1 upright epifluorescence microscope with AxioVision LE 4.6 image acquisition software. Primary antibodies used for IF were anti-53BP1 (H300; Santa Cruz Biotechnology), anti-DNA-PKcs pS2056 (ab18192; Abcam), anti-FANCA (ABP6201; Cascade), anti-FANCD2 (NB100–182; Novus Biologicals and sc-20022; Santa Cruz Biotechnology), anti-FANCI (A300-212A; Bethyl Laboratories), anti-FANCM (Meetei and Deans CE56.1 antibodies), anti-FK2 (sc-8017; Santa Cruz Biotechnology), and anti-γH2AX (05–636; Millipore).

### Statistical analysis

Error bars represent standard errors of the means from three independent experiments. *P* values were calculated using a two-tailed Student’s t-test.

## Additional Information

**How to cite this article**: Vuono, E. A. *et al.* The PTEN phosphatase functions cooperatively with the Fanconi anemia proteins in DNA crosslink repair. *Sci. Rep.* **6**, 36439; doi: 10.1038/srep36439 (2016).

**Publisher’s note:** Springer Nature remains neutral with regard to jurisdictional claims in published maps and institutional affiliations.

## Supplementary Material

Supplementary Information
