# Supplementary material for: The PTEN phosphatase functions cooperatively with the Fanconi anemia proteins in DNA crosslink repair
Source: Sci Rep. 2016 Nov 7;6:36439. doi: 10.1038/srep36439 (PMC5098254; doi:10.1038/srep36439)
Supplement: Supplementary Information [file srep36439-s1.pdf]

The PTEN phosphatase functions cooperatively with the Fanconi anemia proteins in DNA crosslink repair

Elizabeth A. Vuono,[1] Ananda Mukherjee,[2] David A. Vierra,[1] Morganne M. Adroved,[1] Charlotte Hodson,[3] Andrew J. Deans,[3,4] and Niall G. Howlett[1,*]

[1]Department of Cell and Molecular Biology, University of Rhode Island, Kingston, Rhode Island, U.S.A

[2]Department of Obstetrics, Gynecology, and Reproductive Biology, Michigan State University, Grand Rapids, Michigan, U.S.A.

[3]Genome Stability Unit, St. Vincent's Institute, Fitzroy, VIC 3065, Australia

[4]Department of Medicine, The University of Melbourne, Parkville, VIC 3010, Australia

*To whom correspondence should be addressed: Niall G. Howlett Ph.D., 379 Center for Biotechnology and Life Sciences, 120 Flagg Road, Kingston, RI, USA, Tel.: +1 401 874 4306; Fax: +1 401 874 2065; *Email address*: nhowlett@uri.edu

Supplementary information

Materials and methods

Antibody generation

FANCM monoclonal antibodies were generated by intra-peritoneal injection of recombinant purified FANCM peptide (His-tagged amino acids 1507-1647 purified from *E. coli* using Nickel-NTA resin under guanidine-HCl denaturing conditions). Standard hybridoma generation protocols were employed using Sp2/0-Ag14 murine myeloma cells, followed by single cell cloning of the highest titer lines. Independently derived monoclonal antibodies CE56.1, CP3.2, CV5.1, and CV11.1 were purified from hybridoma supernatants using protein A sepharose, and shown to detect a single band of 230 kDa by Western blotting, that is absent in FANCM siRNA treated cells (see **Figure S10**).

Plasmids and site-directed mutagenesis

The plasmids pSG5L-HA-PTEN wild type, -PTEN-C124S, -PTEN-G129E and pHR-SIN-PTEN-Y138L (Addgene) were used to amplify *PTEN* cDNA using the forward primer 5’-CACCATGACAGCCATCATCAAA-3’ and the reverse primer 5’-TCAGACTTTTGTAATTTGTGTATGCTG-3’. The cDNAs were then recombined into pLenti6.2/V5-DEST (Invitrogen) using Invitrogen Gateway Cloning**.** The *PTEN-K254R* cDNA was generated by site-directed mutagenesis of the wild type *PTEN* cDNA using the Quikchange Site-directed Mutagenesis Kit (Stratagene). The forward and reverse oligonucleotide sequences used are as follows:

FP 5’-CGTTACCTGTGTGTGGTGATATCCGAGTAGAGTTCTTCCACAAAC-3’

RP 5’-GTTTGTGGAAGAACTCTACTCGGATATCACCACACACAGGTAACG-3’.

Cellular fractionation

Soluble proteins were removed by extraction in cytoskeletal buffer (CSK) (10 mM PIPES pH 6.8, 300 mM sucrose, 100 mM NaCl, 3 mM $MgCl_2$, 1 mM EGTA, and 0.5% v/v Triton-X-100) for 10 minutes at 4^oC. Pellets were washed once with CSK buffer, lysed in SDS sample buffer (2% w/v SDS, 50 mM Tris-HCl pH 7.4, 10 mM EDTA), boiled for 15 min, followed by sonication for 10 s at 10% amplitude using a Fisher Scientific Model 500 Ultrasonic Dismembrator.

Chromosome breakage and cytotoxicity assays

For chromosome breakage assays, cells were incubated in the absence or presence of MMC for 18-24 h. Prior to harvesting, cells were treated with 0.1 µg/ml Colcemid (Gibco/Invitrogen) for 2 h. Cell pellets were incubated in 0.075 M KCl at 37°C for 18 min, followed by fixation in Carnoy's fixative (3:1 methanol:glacial acetic acid) with multiple changes. Cells were dropped onto chilled slides and air-dried prior to staining with 2.5% w/v Giemsa solution (Sigma). Metaphases were analyzed using a Zeiss AxioImager.A1 upright epifluorescent microscope with AxioVision LE 4.6 image acquisition software. For the cell proliferation assay, cells were plated at 10,000 cells/well, treated in the absence or presence of various concentrations of MMC for 48 h and the CellTiter 96® AQ_{ueous} One Solution Reagent (Promega) was added directly to cultured cells, incubated for 2 h and the absorbance at 490 nm was measured with a 96-well Bio-Rad 680 microplate reader. For the MMC survival assay, cells were plated at a density of 10,000 cells/well in 6-well dishes and treated with MMC the following day for 7 days. Cells were then fixed with 10% v/v methanol and 10% v/v acetic acid, stained with 1% w/v crystal violet in methanol, extracted with 0.02% SDS in methanol, and the absorbance at 595 nm was measured

with a 96-well Bio-Rad 680 microplate reader.

***In vitro* phosphatase assays**

Recombinant FANCM and PTEN were purified as previously described[1,2]. 20 µL reactions containing FANCM (0.2 µg) and/or PLK1 (Invitrogen, 0.2 µg) were incubated with 50 µM ^{32}P-ATP in reaction buffer (50 mM Tris, pH7.5, 10 mM $MgCl_2$, 2 mM EDTA, 1 mM TCEP) for 30 min. PTEN (0, 0.1, 0.3, 1 µg) was added together with PLK inhibitor BI-2356 (to stop additional phosphorylation from occurring and ensure only dephosphorylation was being measured) followed by another 30 min at 30°C. Then sample-loading buffer (Invitrogen) was added to stop the reaction. After SDS-PAGE, the gel was stained with Instant BLUE (Expedeon), dried and exposed to radiographic film.

siRNA experiments

Cells were plated in six-well dishes at a density of 200,000 cells/well and transfected the following day with PTEN-specific (siPTEN), FANCD2-specific (siFANCD2), or control, non-targeting siRNA (siCtrl) using Lipofectamine 2000 (Invitrogen). Seventy-two hours following transfection, cells were incubated in the absence or presence of MMC and harvested for cytogenetic analysis as described above. The following sense and anti-sense sequences were used: siFANCD2 (Dharmacon) 5’-CAGCCUACCUGAGAUCCUA-3’ and 5’-UAGGAUCUCAGGUAGGCUG-3’ and for non-targeting siCtrl (Dharmacon) 5’-UAACGACGCGACGACGUAA-3’ and 5’-UUACGUCGUCGCGUCGUUA-3’. PTEN siRNAs (siGENOME SMARTpool siRNA reagent D-001810-01-05) were obtained from Thermo Fisher Scientific.

Mitomycin C

The concentrations of mitomycin C (MMC) used in this study vary widely depending on the assay being performed. Lower concentrations of MMC (10-40 nM) are used for metaphase chromosome analyses, as higher concentrations will arrest cells in M-phase, resulting in a low yield of mitotic cells, precluding analysis. In contrast, when using immunoblotting or immunofluorescence microscopy to examine the activation of signal transduction pathways, higher concentrations of MMC (>200 nM) are typically necessary to detect protein posttranslational modifications. For cell survival assays, a wide range of concentrations (10 nM - 1 μM) is typically employed.

Figure legends

Figure S1. PTEN$^{-/-}$ cells are hypersensitive to mitomycin C

(A) HCT116 PTEN$^{+/+}$ and two independent clones of PTEN$^{-/-}$ cells were incubated in the absence or presence of the indicated concentrations of MMC for 7-10 days, and cell survival was determined using a crystal violet assay. (B) MCF10A PTEN$^{+/+}$ and PTEN$^{-/-}$ cells were incubated in the absence or presence of the indicated concentrations of MMC for 7-10 days and cell survival determined. (C and D) MCF10A PTEN$^{+/+}$ and PTEN$^{-/-}$ cells were incubated in the absence or presence of the indicated concentrations of MMC for 24 h and metaphase spreads were analyzed for chromosome aberrations. (C) Numbers of centromere aberrations observed in PTEN$^{+/+}$ and PTEN$^{-/-}$ cells and (D) representative images of centromere aberrations observed in PTEN$^{-/-}$ cells. ***, $P < 0.001$.

Figure S2. PTEN is required for efficient mitomycin C-inducible FANCD2 and FANCI nuclear foci formation, but not for the localization of FANCD2 and FANCI to chromatin

(A) Chromatin fractionation analysis of lysates from HCT116 $PTEN^{+/+}$ and $PTEN^{-/-}$ cells reveals efficient localization of FANCD2, FANCI, and RAD51 to chromatin following mitomycin C (MMC) treatment. Cells were incubated in the absence (NT) or presence of 200 nM MMC for 24 h. W, unfractionated whole-cell lysate; S, soluble cytoplasmic and nuclear fraction; C, chromatin-enriched fraction. (B and C) MCF10A $PTEN^{+/+}$ and $PTEN^{-/-}$ cells were incubated in the absence or presence of 200 nM MMC for 18 h, allowed to recover for 8 h, and FANCD2 (B) and FANCI (C) nuclear foci formation was analyzed by immunofluorescence microscopy (IF). Quantification of the percentage of $PTEN^{+/+}$ and $PTEN^{-/-}$ nuclei displaying greater than five discrete FANCD2 or FANCI nuclear foci reveals defective MMC-inducible FANCD2 and FANCI foci formation in $PTEN^{-/-}$ cells. ***, $P < 0.001$. (D) Immunoblotting for FANCD2, FANCI, and RAD51 reveals no appreciable differences in total cellular levels of MMC-inducible FANCD2 and FANCI monoubiquitination or RAD51 between MCF10A $PTEN^{+/+}$ and $PTEN^{-/-}$ cells. Cells were incubated in the absence or presence of 200 nM MMC for 24 h and allowed to recover for 8 h. (E) Chromatin fractionation analysis of lysates from MCF10A $PTEN^{+/+}$ and $PTEN^{-/-}$ cells reveals efficient localization of FANCD2, FANCI, and RAD51 to chromatin following MMC treatment. Cells were incubated in the absence (NT) or presence of 200 nM MMC for 24 h. W, unfractionated whole-cell lysate; S, soluble cytoplasmic and nuclear fraction; C, chromatin-enriched fraction. Immunoblotting experiments were performed multiple times with similar results. Protein band quantifications are from the immunoblots shown and are representative of results from several experiments.

Figure S3. Increased FANCM instability and defective chromatin recruitment of the FA core complex in PTEN-deficient cells

(A) Immunoblotting analyses of whole-cell lysates from HCT116 PTEN$^{+/+}$ and PTEN$^{-/-}$ cells reveal decreased levels of FANCM in PTEN$^{-/-}$ cells. Cells were incubated in the absence (NT) or presence of 40 nM mitomycin C (MMC) for 18 h and allowed to recover for up to 24 h. (B) Immunoblotting analyses of whole-cell lysates from MCF10A PTEN$^{+/+}$ and PTEN$^{-/-}$ cells reveal decreased levels of FANCM in PTEN$^{-/-}$ cells. Cells were treated as described for (A). RBI, Relative protein band intensity with respect to lane 1. (C) MCF10A PTEN$^{+/+}$ and PTEN$^{-/-}$ cells were incubated in the absence or presence of 40 μg/mL cycloheximide (CHX) alone or 40 μg/mL CHX and 4 μM MG132 (CHX + MG) for the indicated times. Whole-cell lysates were prepared and immunoblotted with anti-FANCM, anti-p53, anti-PTEN, and anti-α-tubulin antibodies. RBI, Relative protein band intensity with respect to the untreated sample lane. (D) Quantification of FANCM nuclear foci formation in HCT116 PTEN$^{+/+}$ and PTEN$^{-/-}$ cells reveals a defect in MMC-inducible FANCM nuclear foci formation in PTEN$^{-/-}$ cells. Cells were incubated in the absence (NT) or presence of 40 nM MMC for 18 h. ***, $P < 0.001$. (E) Quantification of FANCA nuclear foci formation in HCT116 PTEN$^{+/+}$ and PTEN$^{-/-}$ cells reveals reduced MMC-inducible FANCA nuclear foci formation in PTEN$^{-/-}$ cells. Cells were treated as described for (D). ***, $P < 0.001$. (F) Chromatin fractionation analysis of HCT116 PTEN$^{+/+}$ and PTEN$^{-/-}$ cells reveals a defect in the localization of FANCA to chromatin following MMC treatment. Cells were incubated in the absence (NT) or presence of 200 nM MMC for 24 h. W, unfractionated whole-cell lysate; S, soluble cytoplasmic and nuclear fraction; C, chromatin fraction. C:W, Ratio of protein in the chromatin fraction versus the whole-cell lysate. For (A-C and F), to improve clarity and conciseness, the presented blots have been cropped. (G) Defective

MMC-inducible FANCM nuclear foci formation in HCT116 $PTEN^{-/-}$ cells. Cells were treated as described for (D). (H) Defective MMC-inducible FANCA nuclear foci formation in HCT116 $PTEN^{-/-}$ cells. Cells were treated as described for (D). Immunoblotting experiments were performed multiple times with similar results. Protein band quantifications are from the immunoblots shown and are representative of results from several experiments.

Figure S4. PTEN does not dephosphorylate PLK1-phosphorylated FANCM *in vitro*

FANCM was incubated in the presence of PLK1, ^{32}P-ATP and increasing concentrations of PTEN or PTEN-C124S (lipid and protein phosphatase defective). SDS-PAGE was used to separate the proteins and phosphorylation levels were detected by exposing the dried gel to radiographic film. Lanes 5-7 show the input proteins in isolation. *, Indicates some minor contaminants within protein preparations, in particular a minor band in PLK1 preparations runs at the same size as PTEN and is phosphorylated by PLK1 even in the absence of PTEN addition. Some PLK1 autophosphorylation also occurs.

Figure S5. Increased γH2AX and 53BP1 nuclear foci formation in PTEN-deficient cells

(A) Quantification of γH2AX nuclear foci formation in MCF10A $PTEN^{+/+}$ and $PTEN^{-/-}$ cells reveals constitutively elevated and persistent γH2AX nuclear foci in $PTEN^{-/-}$ cells. Cells were incubated in the absence (NT) or presence of 200 nM MMC for 18 h and allowed to recover for up to 24 h. ***, $P < 0.001$. (B) Quantification of 53BP1 nuclear foci formation in $PTEN^{+/+}$ and $PTEN^{-/-}$ cells reveals elevated levels of 53BP1 nuclear foci in $PTEN^{-/-}$ cells with and without MMC treatment. Cells were treated as described for (A). *, $P < 0.05$; **, $P < 0.01$; ***, $P <$ 0.001. (C) Chromatin fractionation analysis of HCT116 $PTEN^{+/+}$ and $PTEN^{-/-}$ cells reveals

increased 53BP1 chromatin localization in $PTEN^{-/-}$ cells with and without MMC treatment. Cells were incubated in the absence (NT) or presence of 40 nM MMC for 18 h. W, unfractionated whole-cell lysate; S, soluble cytoplasmic and nuclear fraction; C, chromatin fraction. (D and E) Quantification of RAD51 nuclear foci formation in HCT116 (D) and MCF10A (E) $PTEN^{+/+}$ and $PTEN^{-/-}$ cells reveals no appreciable differences in the levels of RAD51 nuclear foci formation between $PTEN^{+/+}$ and $PTEN^{-/-}$ cells. Cells were incubated in the absence (NT) or presence of 40 nM (HCT116) or 200 nM (MCF10A) MMC for 18 h and allowed to recover for up to 24 h (E).

Figure S6. PTEN function in ICL repair is protein phosphatase and SUMOylation-dependent

(A) HCT116 $PTEN^{-/-}$ cells were stably transduced with pLenti6.2-LacZ, -PTEN-WT, -PTEN-C124S, -PTEN-G129E, -PTEN-K254R, and -PTEN-Y138L. Whole-cell lysates were prepared and immunoblotted for PTEN, AKT, AKT pS473, RAD51, and α-tubulin. (B) PTEN-Y138L fails to rescue the sensitivity of $PTEN^{-/-}$ cells to the clastogenic effects of mitomycin C (MMC), in contrast to PTEN-WT. Cells were incubated in the absence (NT) or presence of 20 nM MMC for 24 h and metaphase spreads were analyzed for structural chromosome aberrations. ***, $P < 0.001$. (C and D) Defective FANCD2 (C) and FANCI (D) nuclear foci formation in $PTEN^{-/-}$ cells expressing PTEN-Y138L. Cells were incubated in the absence (NT) or presence of 200 nM MMC for 18 h. ***, $P < 0.001$. (E and F) Defective FANCA (E) and FANCM (F) nuclear foci formation in $PTEN^{-/-}$ cells expressing LacZ, PTEN-C124S, PTEN-K254R, and PTEN-Y138L. Cells were incubated in the absence (NT) or presence of 200 nM MMC for 18 h. ***, $P < 0.001$.

Figure S7. PTEN SUMOylation promotes its efficient chromatin removal

(A) HCT116 PTEN$^{-/-}$ cells stably transduced with pLenti6.2-PTEN-WT and -PTEN-K254R were incubated in the absence (NT) or presence of 200 nM MMC for 24 h. W, unfractionated whole-cell lysate; S, soluble cytoplasmic and nuclear fraction; C, chromatin fraction. Fractions were immunoblotted for PTEN, H2A, and α-tubulin. C:W, ratio of chromatin-bound protein to total cellular protein. (B) MCF10A PTEN$^{-/-}$ cells stably transduced with pLenti6.2-PTEN-WT and -PTEN-K254R were incubated in the absence (NT) or presence of 200 nM MMC for 24 h, and a cellular fractionation protocol was performed as described in (A). Immunoblotting experiments were performed multiple times with similar results. Protein band quantifications are from the immunoblots shown and are representative of results from several experiments.

Figure S8. FANCD2 and PTEN epistasis analysis

(A) Metaphase chromosome analysis of FA-D2 (*FANCD2*$^{-/-}$) cells reveals that knockdown of PTEN (siPTEN) does not lead to a further increase in the levels of complex chromosome aberrations. siCtrl, control, non-targeting siRNA. (B) siRNA-mediated knockdown of FANCD2 in the PTEN-deficient prostate adenocarcinoma cell line PC-3. (C) Knockdown of FANCD2 (siFANCD2) in PC-3 cells does not lead to a further increase in sensitivity to MMC cytotoxicity. siCtrl, control, non-targeting siRNA. (D-F) Metaphase chromosome analysis of PC-3 cells reveals that knockdown of FANCD2 does not lead to a further increase in the levels of chromosome gaps and breaks (D), complex chromosome aberrations (E), or total chromosome aberrations (F).

Figure S9. Proposed model of the role of PTEN in ICL repair

PTEN has previously been shown to be required for the assembly of APC/C-CDH1 and the degradation of PLK1[3]. PLK1 directly phosphorylates PTEN, promoting its accumulation in chromatin[4]. In turn, PTEN dephosphorylates PLK1, leading to its inactivation and destabilization[5]. When PTEN is present, upon DNA damage, it dephosphorylates PLK1, which allows FANCM to promote the chromatin recruitment of FANCA and the FA core complex, resulting in efficient FANCD2 and FANCI nuclear foci formation and error-free ICL repair. In the absence of PTEN, PLK1 fails to be dephosphorylated, remains constitutively active and phosphorylates FANCM. FANCM phosphorylation leads to its ubiquitination and subsequent degradation by the proteasome. The FANCM phosphatase remains to be identified. When FANCM levels are low, the chromatin recruitment of FANCA and the FA core complex is attenuated, leading to inefficient FANCD2 and FANCI nuclear foci formation and error-prone ICL repair. A, FANCA; D2, FANCD2; I, FANCI; M, FANCM; P, phosphorylation; Ub, ubiquitination. Black- and grey-filled arrows indicate functional and non-functional steps, respectively.

Figure S10. Validation of the FANCM mouse monoclonal antibodies used in these studies

293 cells were transfected with control non-targeting siRNA (lane 1) or siRNA targeting FANCM (lane 2). Whole-cell extracts were prepared in 10 mM Tris, 150 mM NaCl, 10%v/v glycerol, 0.1%w/v Triton X-100, 2 U/mL benzonase. Proteins were resolved on NuPAGE 3-8% w/v Tris-Acetate gels (Invitrogen), transferred to polyvinylidene difluoride (PVDF) membranes, and immunoblotted with the indicated anti-FANCM mouse monoclonal antibodies.

References

1 Chia, Y. C. *et al.* The C-terminal tail inhibitory phosphorylation sites of PTEN regulate its intrinsic catalytic activity and the kinetics of its binding to phosphatidylinositol-4,5-bisphosphate. *Arch Biochem Biophys* **587**, 48-60 (2015).

2 Coulthard, R. *et al.* Architecture and DNA recognition elements of the Fanconi anemia FANCM-FAAP24 complex. *Structure* **21**, 1648-1658 (2013).

3 Song, M. S. *et al.* Nuclear PTEN regulates the APC-CDH1 tumor-suppressive complex in a phosphatase-independent manner. *Cell* **144**, 187-199 (2011).

4 Choi, B. H., Pagano, M. & Dai, W. Plk1 protein phosphorylates phosphatase and tensin homolog (PTEN) and regulates its mitotic activity during the cell cycle. *J Biol Chem* **289**, 14066-14074 (2014).

5 Zhang, Z. *et al.* PTEN regulates PLK1 and controls chromosomal stability during cell division. *Cell Cycle*, 1-10 (2016).

Figure S1

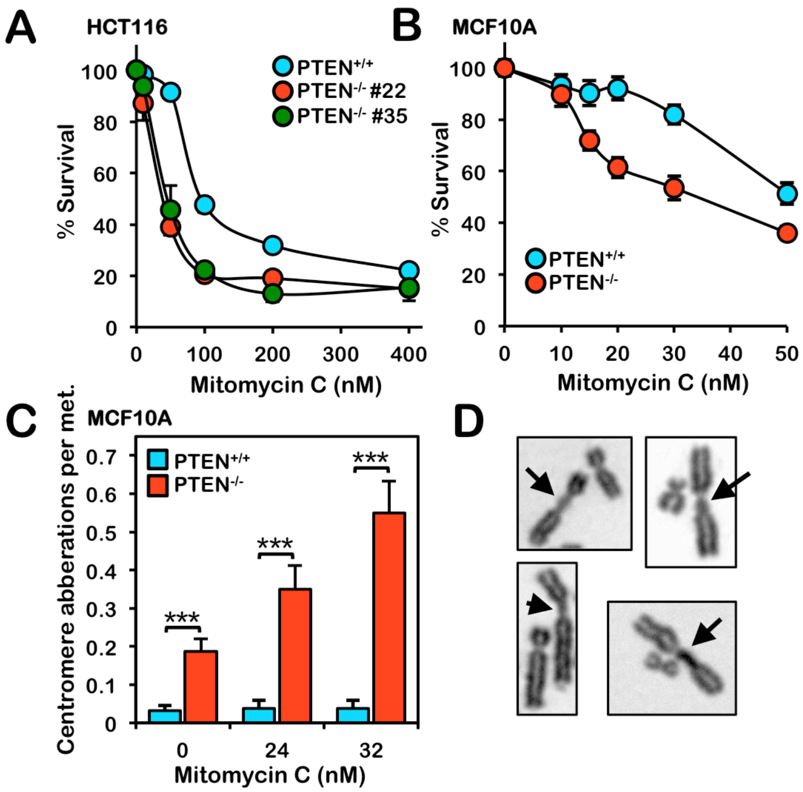

A
HCT116
PTEN+/+
PTEN-/- #22
PTEN-/- #35
% Survival
100
80
60
40
20
0
0
100
200
300
400
Mitomycin C (nM)
B
MCF10A
PTEN+/+
PTEN-/-
% Survival
0
10
20
30
40
50
Mitomycin C (nM)
C
MCF10A
PTEN+/+
PTEN-/-
Centromere abberations per met.
0.7
0.6
0.5
0.4
0.3
0.2
0.1
***
0
24
32
Mitomycin C (nM)
D

Figure S2A-C

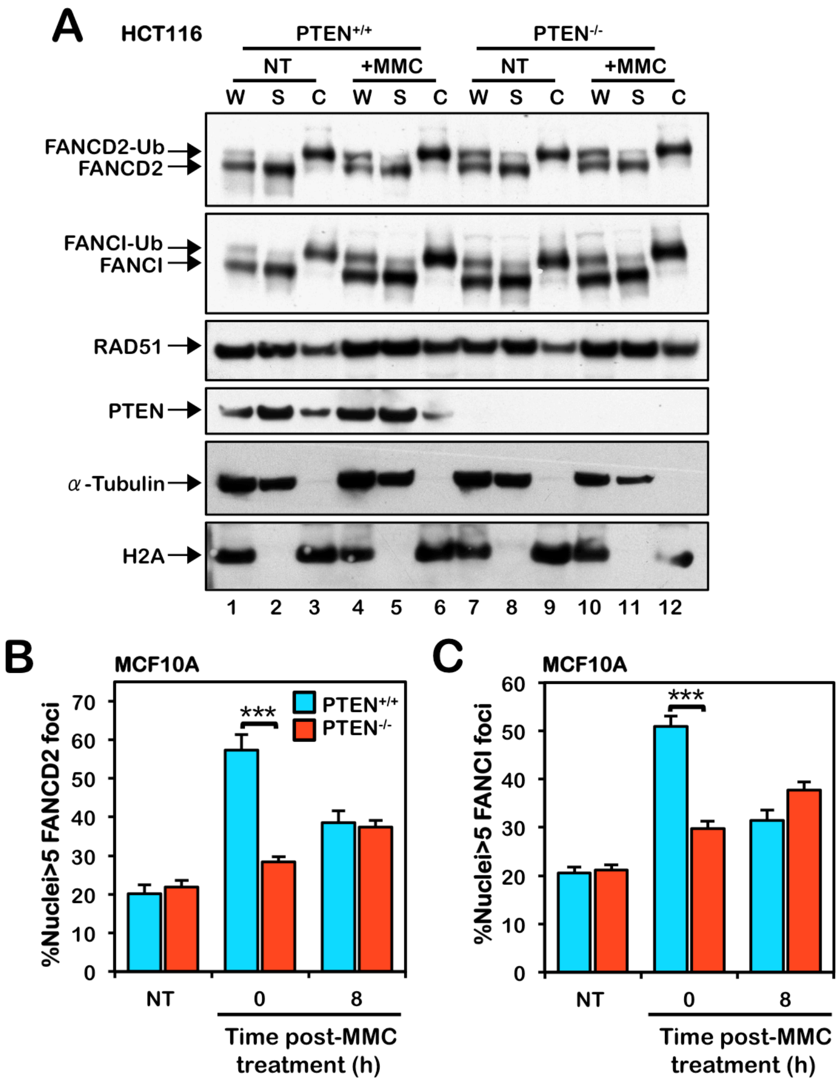
A
HCT116
PTEN+/+
PTEN-/-
NT
+MMC
W S C
FANCD2-Ub
FANCD2
FANCI-Ub
FANCI
RAD51
PTEN
α-Tubulin
H2A
1 2 3 4 5 6 7 8 9 10 11 12
B
MCF10A
%Nuclei>5 FANCD2 foci
PTEN+/+
PTEN-/-
***
NT
0
8
Time post-MMC treatment (h)
C
MCF10A
%Nuclei>5 FANCI foci
***
NT
0
8
Time post-MMC treatment (h)

Figure S2D-E

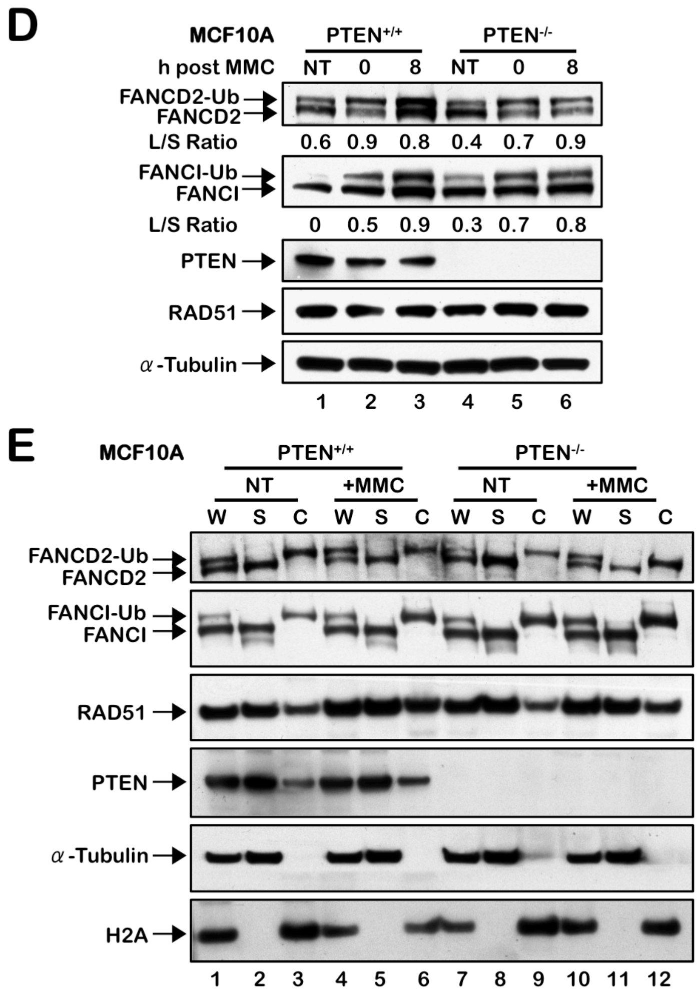
D
MCF10A
PTEN+/+
PTEN-/-
h post MMC
NT 0 8 NT 0 8
FANCD2-Ub
FANCD2
L/S Ratio
0.6 0.9 0.8 0.4 0.7 0.9
FANCI-Ub
FANCI
L/S Ratio
0 0.5 0.9 0.3 0.7 0.8
PTEN
RAD51
α-Tubulin
1 2 3 4 5 6
E
MCF10A
PTEN+/+
PTEN-/-
NT
+MMC
NT
+MMC
W S C W S C W S C W S C
FANCD2-Ub
FANCD2
FANCI-Ub
FANCI
RAD51
PTEN
α-Tubulin
H2A
1 2 3 4 5 6 7 8 9 10 11 12

Figure S3A-C

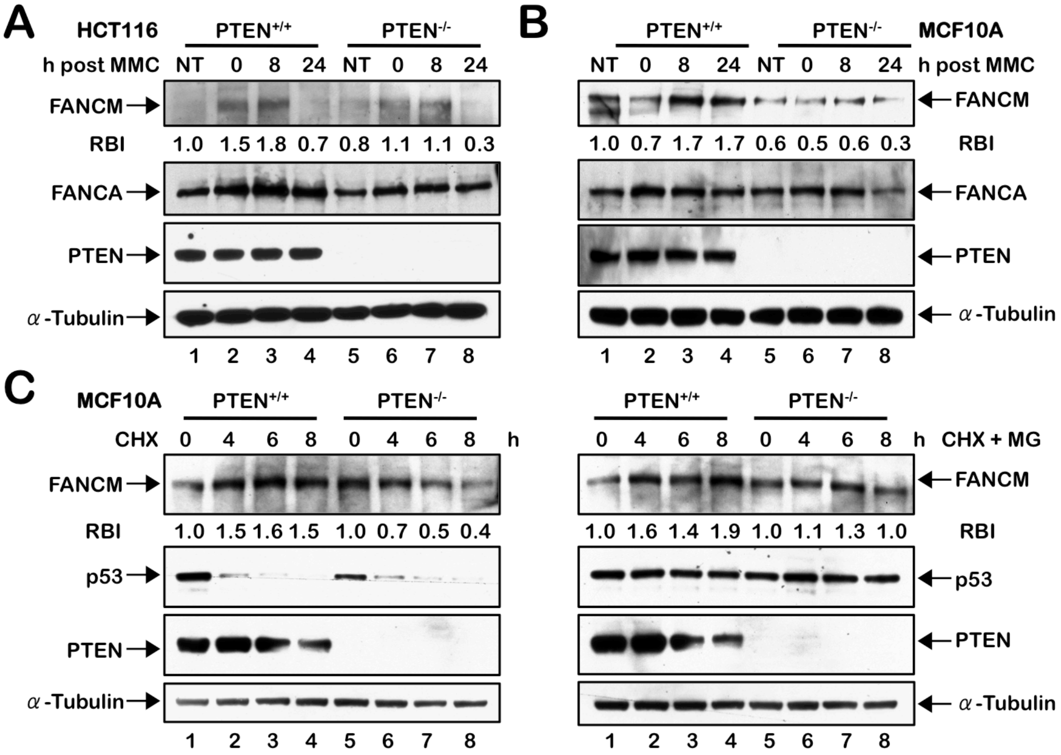
A
HCT116
PTEN+/+
PTEN-/-
h post MMC NT 0 8 24 NT 0 8 24
FANCM
RBI 1.0 1.5 1.8 0.7 0.8 1.1 1.1 0.3
FANCA
PTEN
α-Tubulin
1 2 3 4 5 6 7 8
B
PTEN+/+
PTEN-/-
MCF10A
NT 0 8 24 NT 0 8 24 h post MMC
FANCM
1.0 0.7 1.7 1.7 0.6 0.5 0.6 0.3 RBI
FANCA
PTEN
α-Tubulin
1 2 3 4 5 6 7 8
C
MCF10A
PTEN+/+
PTEN-/-
CHX 0 4 6 8 0 4 6 8 h
FANCM
RBI 1.0 1.5 1.6 1.5 1.0 0.7 0.5 0.4
p53
PTEN
α-Tubulin
1 2 3 4 5 6 7 8
PTEN+/+
PTEN-/-
0 4 6 8 0 4 6 8 h CHX + MG
FANCM
1.0 1.6 1.4 1.9 1.0 1.1 1.3 1.0 RBI
p53
PTEN
α-Tubulin
1 2 3 4 5 6 7 8

Figure S3D-F

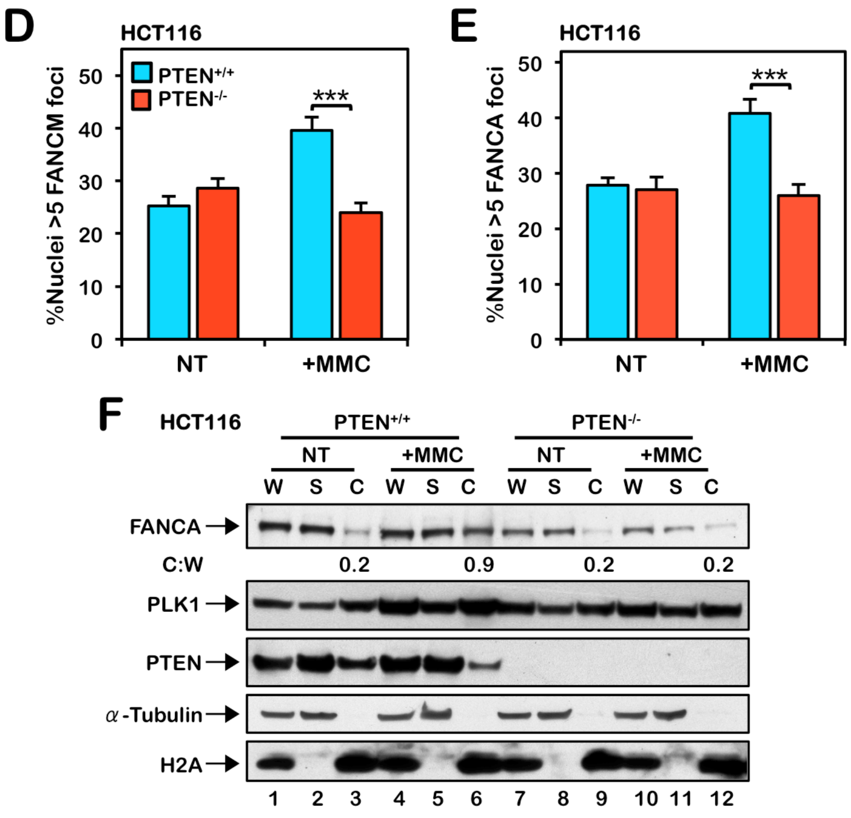
D
HCT116
%Nuclei >5 FANCM foci
PTEN+/+
PTEN-/-
***
0
10
20
30
40
50
NT
+MMC
E
HCT116
%Nuclei >5 FANCA foci
***
NT
+MMC
F
HCT116
PTEN+/+
PTEN-/-
NT
+MMC
W S C W S C W S C W S C
FANCA
C:W
0.2
0.9
0.2
0.2
PLK1
PTEN
α-Tubulin
H2A
1 2 3 4 5 6 7 8 9 10 11 12

Figure S3G

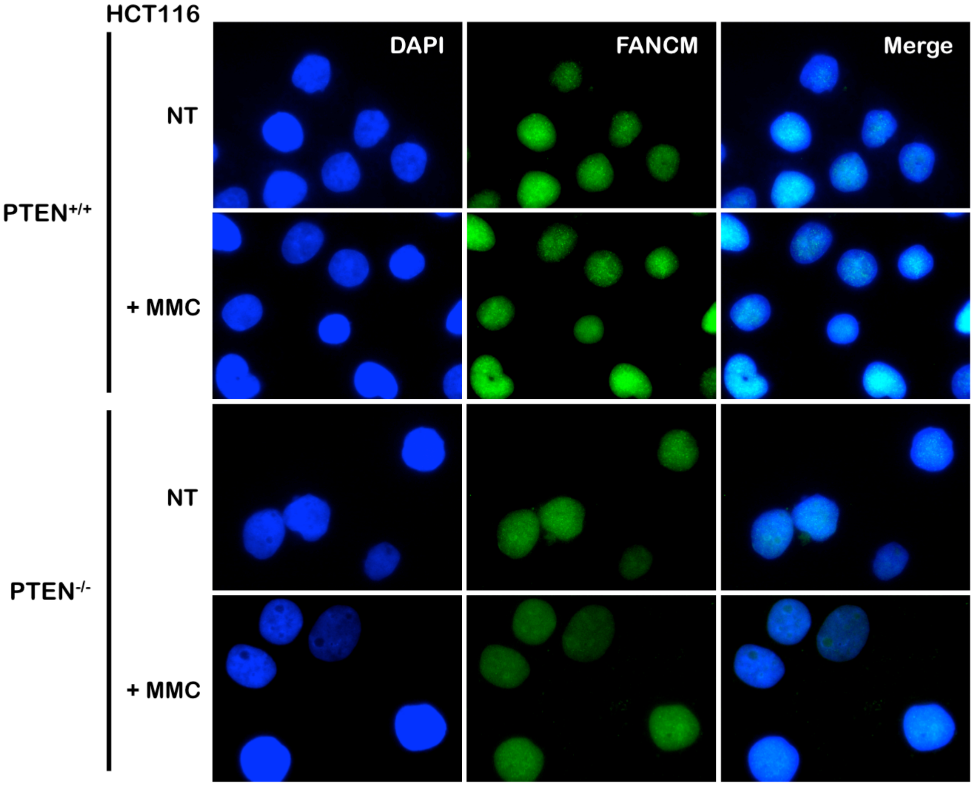

Figure S3H

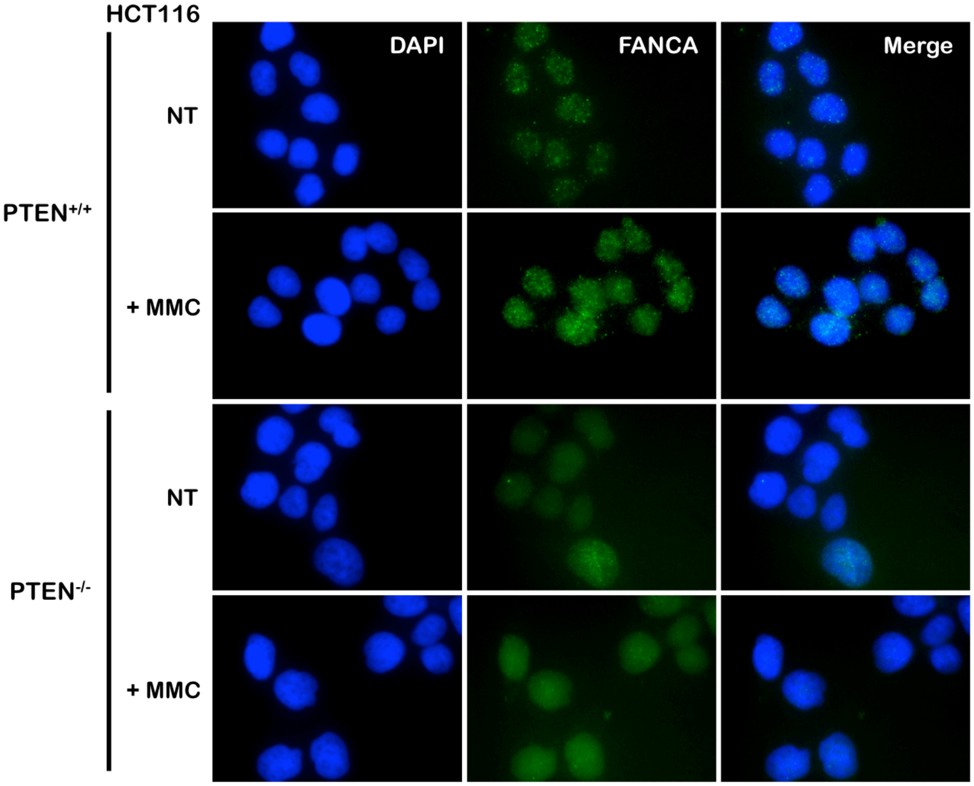

Figure S4

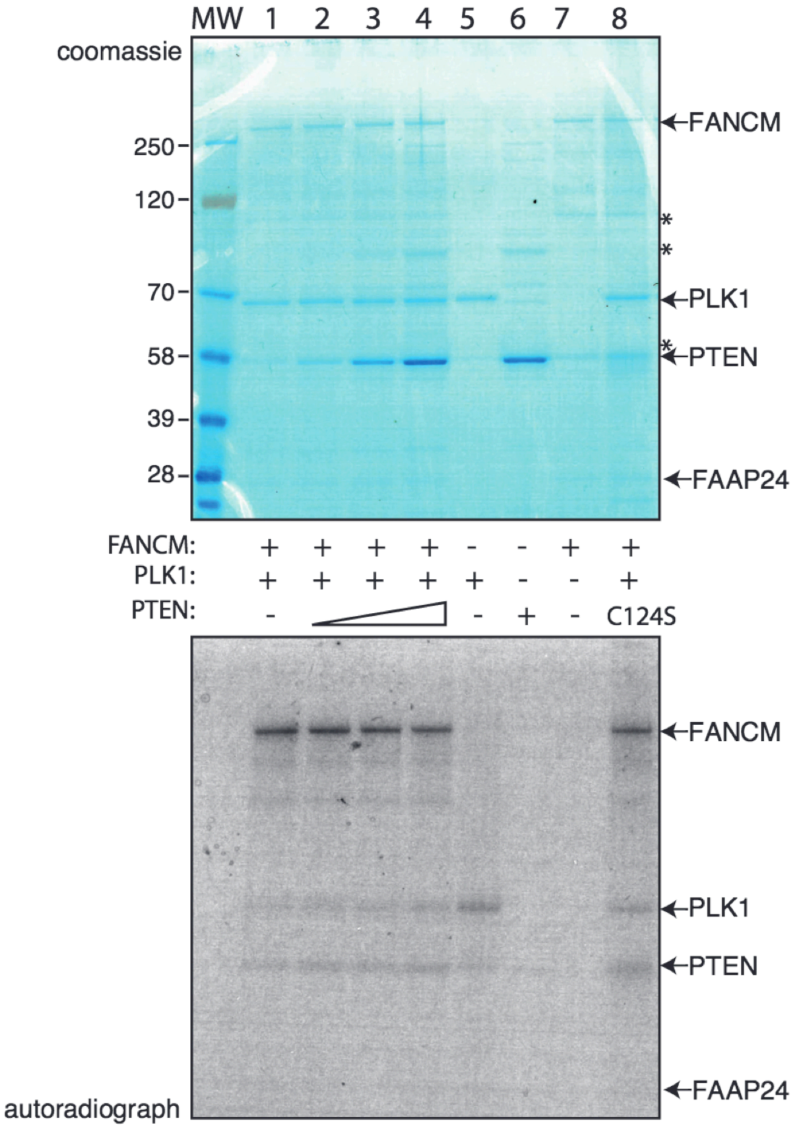

MW 1 2 3 4 5 6 7 8
coomassie
250
120
70
58
39
28
FANCM
*
*
PLK1
*
PTEN
FAAP24
FANCM: + + + + - - + +
PLK1: + + + + + - - +
PTEN: - + - + - C124S
autoradiograph
FANCM
PLK1
PTEN
FAAP24

Figure S5

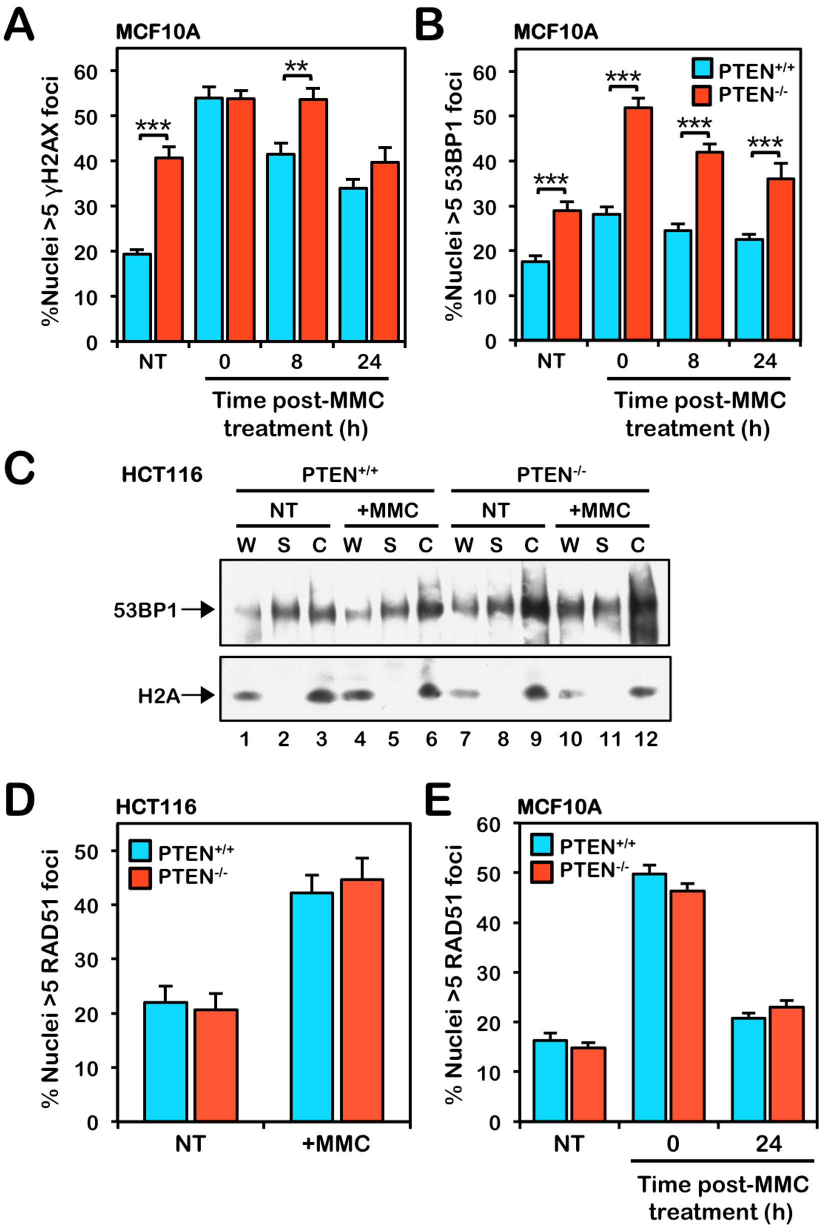
A
MCF10A
%Nuclei >5 γH2AX foci
0 10 20 30 40 50 60
***
**
NT 0 8 24
Time post-MMC treatment (h)
B
MCF10A
%Nuclei >5 53BP1 foci
0 10 20 30 40 50 60
PTEN+/+
PTEN-/-
***
NT 0 8 24
Time post-MMC treatment (h)
C
HCT116
PTEN+/+
PTEN-/-
NT +MMC NT +MMC
W S C W S C W S C W S C
53BP1
H2A
1 2 3 4 5 6 7 8 9 10 11 12
D
HCT116
% Nuclei >5 RAD51 foci
0 10 20 30 40 50
PTEN+/+
PTEN-/-
NT +MMC
E
MCF10A
% Nuclei >5 RAD51 foci
0 10 20 30 40 50 60
PTEN+/+
PTEN-/-
NT 0 24
Time post-MMC treatment (h)

Figure S6A-D

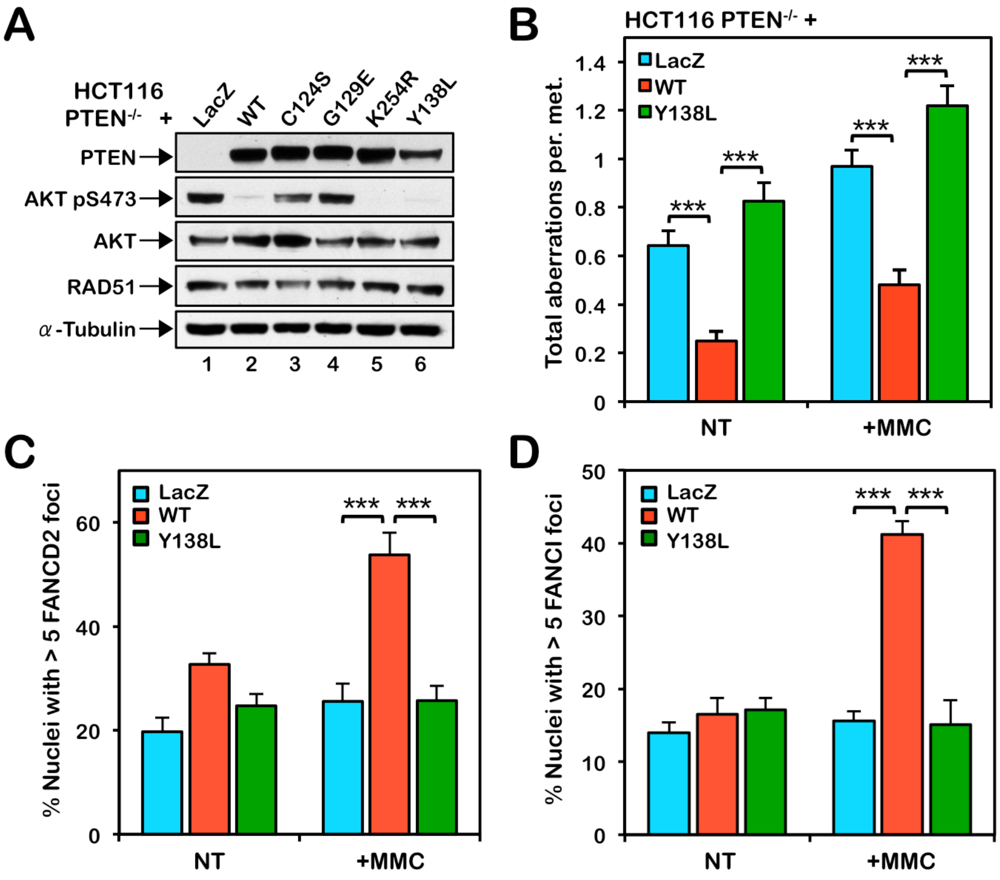
A
HCT116 PTEN-/- +
LacZ
WT
C124S
G129E
K254R
Y138L
PTEN
AKT pS473
AKT
RAD51
α-Tubulin
1 2 3 4 5 6
B
HCT116 PTEN-/- +
Total aberrations per. met.
LacZ
WT
Y138L
***
NT
+MMC
C
% Nuclei with > 5 FANCD2 foci
D
% Nuclei with > 5 FANCI foci

Figure S6E-F

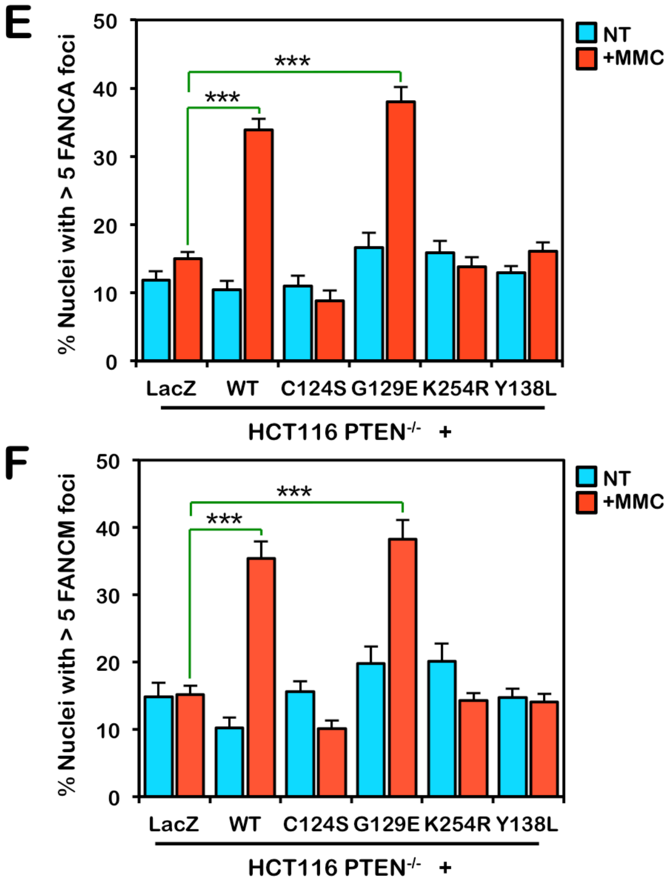

E
50
40
30
20
10
0
% Nuclei with > 5 FANCA foci
NT
+MMC
***
***
LacZ
WT
C124S
G129E
K254R
Y138L
HCT116 PTEN-/- +
F
50
40
30
20
10
0
% Nuclei with > 5 FANCM foci
NT
+MMC
***
***
LacZ
WT
C124S
G129E
K254R
Y138L
HCT116 PTEN-/- +

Figure S7

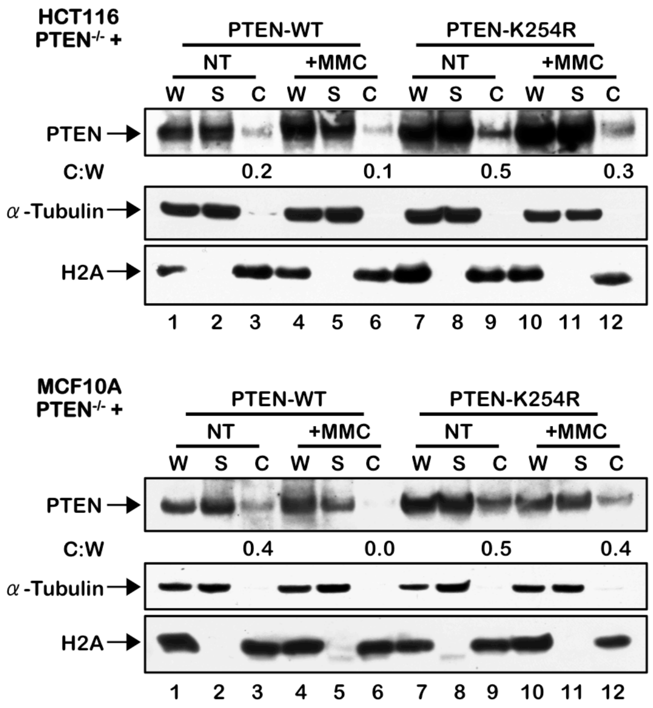
HCT116
PTEN-/- +
PTEN-WT
PTEN-K254R
NT
+MMC
NT
+MMC
W S C W S C W S C W S C
PTEN
C:W
0.2
0.1
0.5
0.3
α-Tubulin
H2A
1 2 3 4 5 6 7 8 9 10 11 12
MCF10A
PTEN-/- +
PTEN-WT
PTEN-K254R
NT
+MMC
NT
+MMC
W S C W S C W S C W S C
PTEN
C:W
0.4
0.0
0.5
0.4
α-Tubulin
H2A
1 2 3 4 5 6 7 8 9 10 11 12

Figure S8

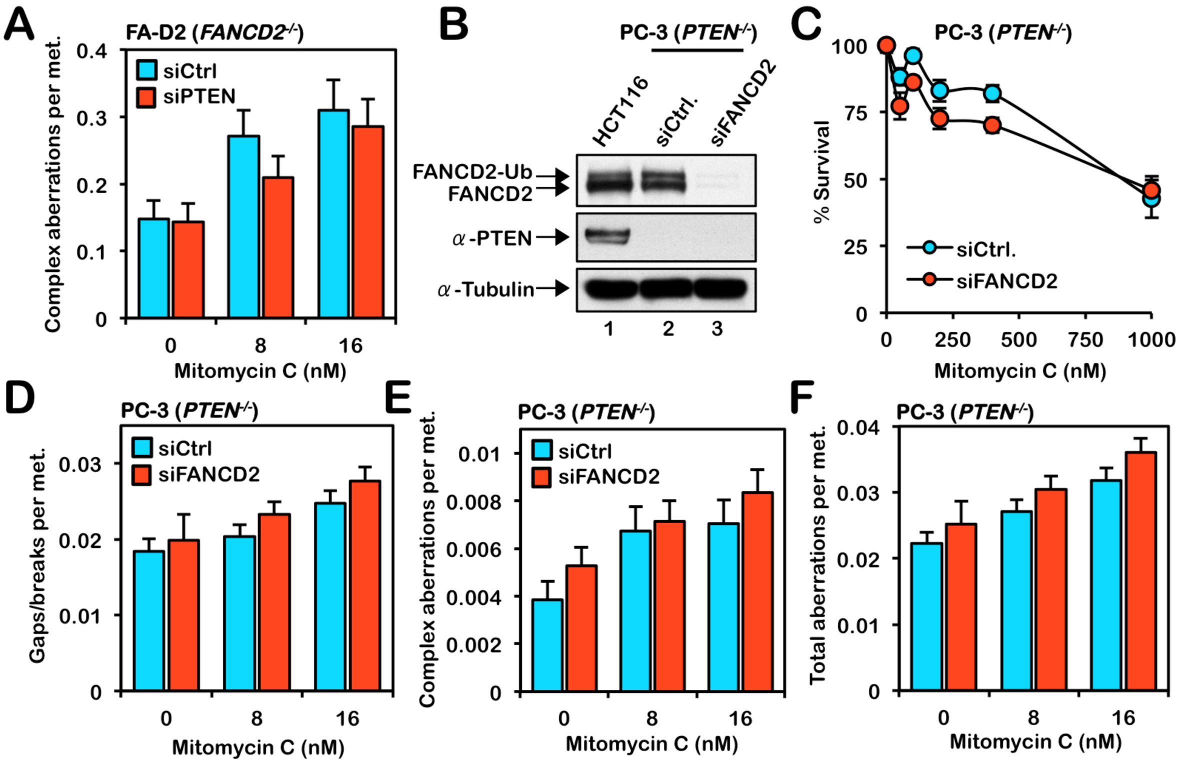

A
FA-D2 (FANCD2-/-)
siCtrl
siPTEN
Complex aberrations per met.
0.4
0.3
0.2
0.1
0
0
8
16
Mitomycin C (nM)
B
PC-3 (PTEN-/-)
HCT116
siCtrl.
siFANCD2
FANCD2-Ub
FANCD2
α-PTEN
α-Tubulin
1
2
3
C
PC-3 (PTEN-/-)
% Survival
100
75
50
25
0
siCtrl.
siFANCD2
0
250
500
750
1000
Mitomycin C (nM)
D
PC-3 (PTEN-/-)
siCtrl
siFANCD2
Gaps/breaks per met.
0.03
0.02
0.01
0
0
8
16
Mitomycin C (nM)
E
PC-3 (PTEN-/-)
siCtrl
siFANCD2
Complex aberrations per met.
0.01
0.008
0.006
0.004
0.002
0
0
8
16
Mitomycin C (nM)
F
PC-3 (PTEN-/-)
Total aberrations per met.
0.04
0.03
0.02
0.01
0
0
8
16
Mitomycin C (nM)

Figure S9

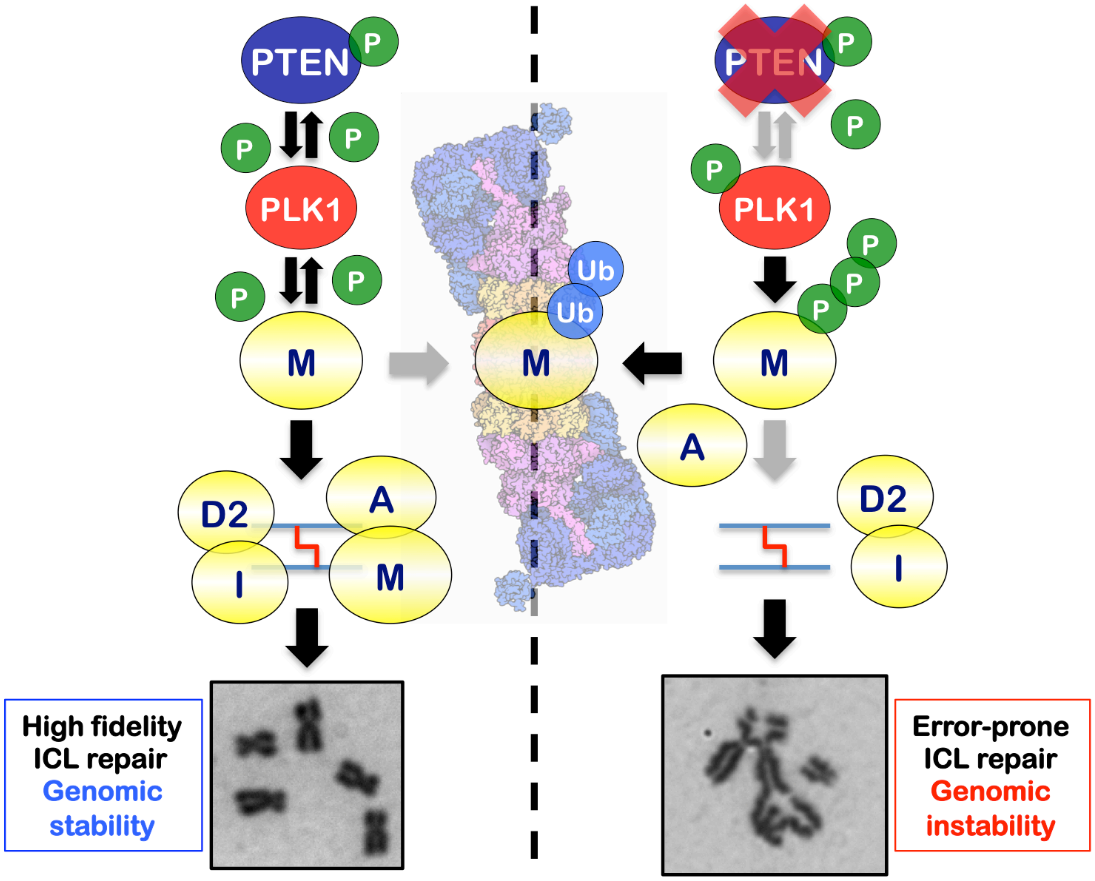

PTEN
P
PLK1
M
Ub
Ub
A
D2
I
High fidelity ICL repair
Genomic stability
Error-prone ICL repair
Genomic instability

Figure S10

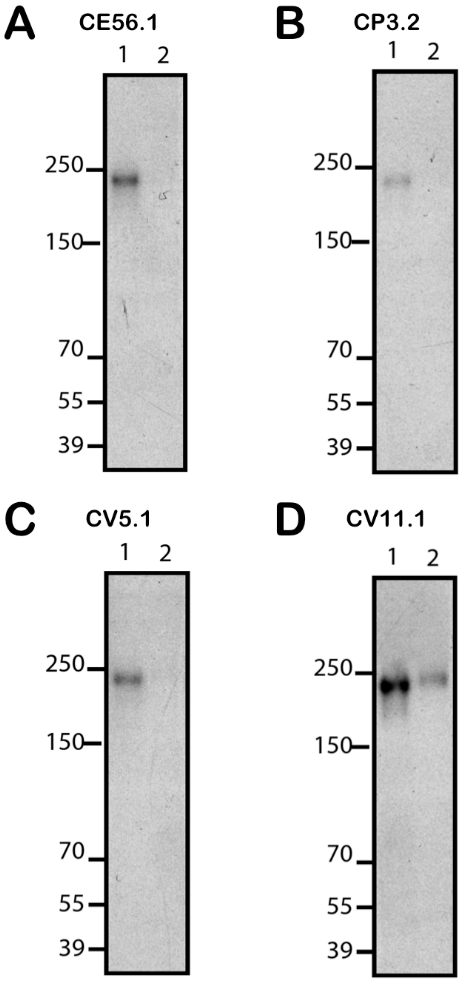
A
CE56.1
1 2
250
150
70
55
39
B
CP3.2
1 2
250
150
70
55
39
C
CV5.1
1 2
250
150
70
55
39
D
CV11.1
1 2
250
150
70
55
39
